# Preventive malaria treatment among school-aged children in sub-Saharan Africa: a systematic review and meta-analyses

**DOI:** 10.1016/S2214-109X(20)30325-9

**Published:** 2020-10-22

**Authors:** Lauren M Cohee, Charles Opondo, Siân E Clarke, Katherine E Halliday, Jorge Cano, Andrea G Shipper, Breanna Barger-Kamate, Abdoulaye Djimde, Seybou Diarra, Aditi Dokras, Moses R Kamya, Pascal Lutumba, Alioune Badara Ly, Joaniter I Nankabirwa, J Kiambo Njagi, Hamma Maiga, Catherine Maiteki-Sebuguzi, Junior Matangila, George Okello, Fabian Rohner, Natalie Roschnik, Saba Rouhani, Mahamadou S Sissoko, Sarah G Staedke, Mahamadou A Thera, Elizabeth L Turner, JP Van Geertruyden, Michael B Zimmerman, Matthew C H Jukes, Simon J Brooker, Elizabeth Allen, Miriam K Laufer, R Matthew Chico

**Affiliations:** Center for Vaccine Development and Global Health, University of Maryland, Baltimore, MA, USA; Department of Medical Statistics, Faculty of Epidemiology and Population Health, Faculty of Infectious and Tropical Diseases, London School of Hygiene & Tropical Medicine, London, UK; Department of Disease Control, Faculty of Infectious and Tropical Diseases, London School of Hygiene & Tropical Medicine, London, UK; Department of Disease Control, Faculty of Infectious and Tropical Diseases, London School of Hygiene & Tropical Medicine, London, UK; Department of Disease Control, Faculty of Infectious and Tropical Diseases, London School of Hygiene & Tropical Medicine, London, UK; University of Maryland School of Medicine, and Health Sciences and Human Services Library, University of Maryland, Baltimore, MA, USA; School of Medicine, University of Washington, Seattle, WA, USA; Faculty of Medicine, Pharmacy, and Odnonto-Stomatology, Malaria Research and Training Center, University of Sciences, Techniques and Technologies of Bamako, Mali; Save the Children, Bamako, Mali; Department of Pediatrics, University of Maryland, Baltimore, MA, USA; Infectious Diseases Research Collaboration, Kampala, Uganda; Tropical Medicine Department, University of Kinshasa, Kinshasa, Democratic Republic of Congo; Ministère de la Santé et de l’Action Sociale, Dakar, Senegal; School of Medicine, Makerere University College of Health Sciences, Kampala, Uganda; Infectious Diseases Research Collaboration, Kampala, Uganda; National Malaria Control Programme, Ministry of Health, Nairobi, Kenya; Faculty of Medicine, Pharmacy, and Odnonto-Stomatology, Malaria Research and Training Center, University of Sciences, Techniques and Technologies of Bamako, Mali; Infectious Diseases Research Collaboration, Kampala, Uganda; Tropical Medicine Department, University of Kinshasa, Kinshasa, Democratic Republic of Congo; Global Health Institute, University of Antwerp, Antwerp, Belgium; Health Systems and Social Science Research Group, Kenya Medical Research Institute-Wellcome Trust Research Programme, Kilifi, Kenya; GroundWork, Fläsch, Switzerland; Programme Quality and Policy Save the Children UK, London, UK; Department of Disease Control, Faculty of Infectious and Tropical Diseases, London School of Hygiene & Tropical Medicine, London, UK; Save the Children, Bamako, Mali; Faculty of Medicine, Pharmacy, and Odnonto-Stomatology, Malaria Research and Training Center, University of Sciences, Techniques and Technologies of Bamako, Mali; Department of Clinical Research, Faculty of Infectious and Tropical Diseases, London School of Hygiene & Tropical Medicine, London, UK; Faculty of Medicine, Pharmacy, and Odnonto-Stomatology, Malaria Research and Training Center, University of Sciences, Techniques and Technologies of Bamako, Mali; Department of Biostatistics & Bioinformatics and Duke Global Health Institute, Duke University, Durham, NC, USA; Global Health Institute, University of Antwerp, Antwerp, Belgium; Institute of Food, Nutrition, and Health, Swiss Federal Institute of Technology, Zurich, Switzerland; RTI International, London, UK; Bill & Melinda Gates Foundation, Seattle, WA, USA; Department of Medical Statistics, Faculty of Epidemiology and Population Health, Faculty of Infectious and Tropical Diseases, London School of Hygiene & Tropical Medicine, London, UK; Center for Vaccine Development and Global Health, University of Maryland, Baltimore, MA, USA; Department of Disease Control, Faculty of Infectious and Tropical Diseases, London School of Hygiene & Tropical Medicine, London, UK

## Abstract

**Background:**

The burden of malaria infection in sub-Saharan Africa among school-aged children aged 5–15 years is underappreciated and represents an important source of human-to-mosquito transmission of *Plasmodium falciparum*. Additional interventions are needed to control and eliminate malaria. We aimed to assess whether preventive treatment of malaria might be an effective means of reducing *P falciparum* infection and anaemia in school-aged children and lowering parasite transmission.

**Methods:**

In this systematic review and two meta-analyses, we searched the online databases PubMed, Embase, Cochrane CENTRAL, and Clinicaltrials.gov for intervention studies published between Jan 1, 1990, and Dec 14, 2018. We included randomised studies that assessed the effect of antimalarial treatment among asymptomatic school-aged children aged 5–15 years in sub-Saharan Africa on prevalence of *P falciparum* infection and anaemia, clinical malaria, and cognitive function. We first extracted data for a study-level meta-analysis, then contacted research groups to request data for an individual participant data meta-analysis. Outcomes of interest included prevalence of *P falciparum* infection detected by microscopy, anaemia (study defined values or haemoglobin less than age-adjusted and sex-adjusted values), clinical malaria (infection and symptoms on the basis of study-specific definitions) during follow-up, and code transmission test scores. We assessed effects by treatment type and duration of time protected, and explored effect modification by transmission setting. For study-level meta-analysis, we calculated risk ratios for binary outcomes and standardised mean differences for continuous outcomes and pooled outcomes using fixed-effect and random-effects models. We used a hierarchical generalised linear model for meta-analysis of individual participant data. This study is registered with PROSPERO, CRD42016030197.

**Findings:**

Of 628 studies identified, 13 were eligible for the study-level meta-analysis (n=16 309). Researchers from 11 studies contributed data on at least one outcome (n=15 658) for an individual participant data meta-analysis. Interventions and study designs were highly heterogeneous; overall risk of bias was low. In the study-level meta-analysis, treatment was associated with reductions in *P falciparum* prevalence (risk ratio [RR] 0·27, 95% CI 0·17–0·44), anaemia (0·77, 0·65–0·91), and clinical malaria (0·40, 0·28–0·56); results for cognitive outcomes are not presented because data were only available for three trials. In our individual participant data meta-analysis, we found treatment significantly decreased *P falciparum* prevalence (adjusted RR [ARR] 0·46, 95% CI 0·40–0·53; p<0·0001; 15 648 individuals; 11 studies), anaemia (ARR 0·85, 0·77–0·92; p<0·0001; 15 026 individuals; 11 studies), and subsequent clinical malaria (ARR 0·50, 0·39–0·60; p<0·0001; 1815 individuals; four studies) across transmission settings. We detected a marginal effect on cognitive function in children older than 10 years (adjusted mean difference in standardised test scores 0·36, 0·01–0·71; p=0·044; 3962 individuals; five studies) although we found no significant effect when combined across all ages.

**Interpretation:**

Preventive treatment of malaria among school-aged children significantly decreases *P falciparum* prevalence, anaemia, and risk of subsequent clinical malaria across transmission settings. Policy makers and programme managers should consider preventive treatment of malaria to protect this age group and advance the goal of malaria elimination, while weighing these benefits against potential risks of chemoprevention.

## Introduction

Over the last 15 years, increases in access to malaria control interventions have resulted in remarkable declines in malaria-attributable morbidity and mortality. However, since 2014 progress has slowed and the number of malaria cases has even increased in some countries.^1^ Reports from sub-Saharan Africa suggest that *Plasmodium falciparum* infections are more common among school-age children (ie, those aged approximately aged 5–15 years) than among younger children and adults.^2–9^ 200 million school-age children are at risk of malaria in Africa, in many areas of which the prevalence of infection exceeds 50% in this age group.^10,11^ These infections are associated with compromised health,^12,13^ anaemia,^13^ diminished cognitive function,^14^ and lower educational achievement.^15^ Infections in school-age children are also an important source of human-to-mosquito *P falciparum* infection that drives malaria transmission and undermines malaria elimination efforts.^16–18^ Innovative interventions are urgently needed to protect these children from the consequences of *P falciparum* infection and to reduce the reservoir of *P falciparum* circulating in endemic communities.^9,19^

WHO recommends providing intermittent preventive treatment or chemoprevention to asymptomatic pregnant women,^20^ infants,^21^ and preschool children (younger than 5 years) in some malaria-endemic areas.^22^ There are no recommendations, however, for school-aged children, despite mounting evidence that preventive treatment of malaria among school-aged children decreases *P falciparum* infections, malaria-related anaemia, and improves cognitive performance.^23–31^ We aimed to do two meta-analyses of malaria treatment trials among asymptomatic school-aged children: one drawing on summary study-level data, and the other involving individual participant data that allows for subanalyses of treatment type, frequency of treatment, and intervention strategy. We aimed to use the findings to discuss the effect of school-based preventive treatment on *P falciparum* infection, anaemia, subsequent clinical malaria, and cognitive function, as well as the optimal treatment, regimen, target-age group, and transmission setting.

## Methods

### Search strategy and selection criteria

This systematic review and meta-analyses adhered to PRISMA guidelines. We searched PubMed, Embase, Cochrane CENTRAL, and ClinicalTrials.gov to identify malaria studies in school-aged children (for search terms see appendix p 47–48) published between January 1, 1990, and Dec 14, 2018, that targeted child or adolescent participants. We did not have any language restrictions. Two reviewers used predetermined eligibility criteria to screen records and full texts, while a third reviewer adjudicated if the first two reviewers did not agree. Grey literature was also sought through trial registries and abstract searches. No articles required translation and inclusion criteria for the systematic review and the meta-analysis were the same.

### Data analysis

We extracted study-level data without masking to author or publication, and assessed risk of bias in each study using RevMan 5.2 software. For the study-level meta-analysis, we extracted the number of participants, treatment used, dosing interval, and timing of outcome measurement. We then contacted each research group to request individual-level data, including participant age, sex, treatment group, specific geographical locations of the trial, *P falciparum* infection, anaemia, clinical malaria status during follow-up, and code transmission test scores—a common measure of cognitive function based on sustained auditory attention—at baseline and after treatment. Outcomes of interest included prevalence of *P falciparum* infection detected by microscopy, anaemia (study-defined values or, if the study did not report anaemia as a binary variable, haemoglobin less than age-adjusted and sex-adjusted values),^32^ clinical malaria (infection and symptoms on the basis of study specific definitions) during follow-up, and code transmission test scores.

To evaluate the effect of treatment regimens, we grouped interventions by drug class and pharmacokinetic features: sulfadoxine–pyrimethamine alone, sulfadoxine–pyrimethamine combined with an aminoquinoline (either amodiaquine or piperaquine), sulfadoxine–pyrimethamine plus artesunate, artemisinin-based combination therapy including an aminoquinoline (artesunate–amodiaquine or dihydroartemisinin–piperaquine), and artemether–lumefantrine. We constructed a variable to estimate the proportion of follow-up time protected by treatment for each trial (appendix pp 6, 48) to allow for cross-study comparisons of treatment regimens, frequency of retreatment, and length of time after treatment before outcomes were measured. Briefly, we estimated the follow-up time protected based on the chemoprophylaxis after treatment period of each treatment regimen measured in days and multiplied by treatment rounds. We then calculated the proportion of follow-up time according to the number of days protected by treatment and divided by the number of days between the first dose and outcome measurement. We categorised studies according to proportion of follow-up time protected: low (<20%), intermediate (20% to <50%), and high (≥50%).

To assess whether treatment effect varied by transmission setting, we extracted site-specific and year-specific malaria parasite prevalence estimates from the Malaria Atlas Project for the geocoordinates of each school or cluster midpoint involved in each study.^33^ Malaria Atlas estimates reflect the average prevalence of *P falciparum* infection among children aged 2–10 years (*Pf*PR_2–10_) to within 5 km of any location in sub-Saharan Africa. In studies where fieldwork straddled multiple years, we weighted the Malaria Atlas estimates by the number of months of each year that each contributed to the study. We divided areas into WHO transmission settings based on parasite prevalence and further divided mesoendemic into two categories: low (<10%), low–moderate (10% to <30%), moderate–high (30% to <50%), and high (≥50%).^34^

As a first step in our study-level meta-analysis, we calculated risk (prevalence) ratios for binary outcomes and standardised mean differences for continuous outcomes and pooled these outcomes using using random-effects and fixed-effects models. Counts from cluster-randomised studies were divided by the design effect due to clustering before pooling them with individually randomised studies. Between-study heterogeneity was estimated using the *I*² statistic, and meta-regression was done to determine whether any between-study heterogeneity of effect could be explained by study characteristics, including drug class, region of study, prevalence of *P falciparum* infection according to Malaria Atlas estimates, proportion of follow-up time protected, and study design.

Meta-analysis of the individual participant data involved a hierarchical generalised linear model with logit-link followed by marginal standardisation conditional on zero random effects to estimate the risk ratios for the effect of treatment, allowing for random intercepts across studies, and further within clusters for studies that used a clustered design. Code transmission test scores at endline were standardised by subtracting the baseline mean score and dividing this difference by the baseline standard deviation. We used a hierarchical generalised linear model with Gaussian link and adjusted for repeated observations within clusters, within studies to analyse standardised test scores. This approach allowed us to adjust for study-level and individual-level characteristics that could explain heterogeneity of effect, and to incorporate any residual heterogeneity in the estimate of pooled effects. We did not control for combined interventions (eg, bednet distribution) when the intervention was given to both the intervention and the control groups. For multigroup factorial studies, all groups receiving antimalarial treatment were combined and compared with all groups not receiving antimalarial treatment. Age and sex were independently associated with *P falciparum* infection and anaemia, whereas age, but not sex, was associated with clinical malaria and code transmission test scores (appendix p 7). Thus, for consistency we included age, sex, and transmission setting in our fully adjusted models.

To determine whether the effect of treatment varied by local transmission, we fitted an interaction term between treatment and transmission setting for *P falciparum* infection, anaemia, and clinical malaria. There was insufficient variation in transmission setting to do the same among studies measuring code transmission test scores. We compared the effects of different treatment types with control (placebo or no treatment), and the effect of follow-up time protected by treatment type. Because malaria immunity increases with cumulative exposure to *P falciparum* parasites, we stratified results by age to explore the effect of intervention among children aged 5 years to less than 10 years versus children aged 10 years or older to less than 15 years. Because two of the larger trials^35–37^ fundamentally differed from others, we also did sensitivity analyses excluding these datasets. We used Stata/IC 15 software for all analyses. This study is registered with PROSPERO, CRD42016030197.

### Role of the funding source

The funders of the study had no role in study design, data collection, data analysis, data interpretation, or writing the report. The corresponding author had full access to all the data in the study and had final responsibility for the decision to submit for publication.

## Results

**Figure 1:**
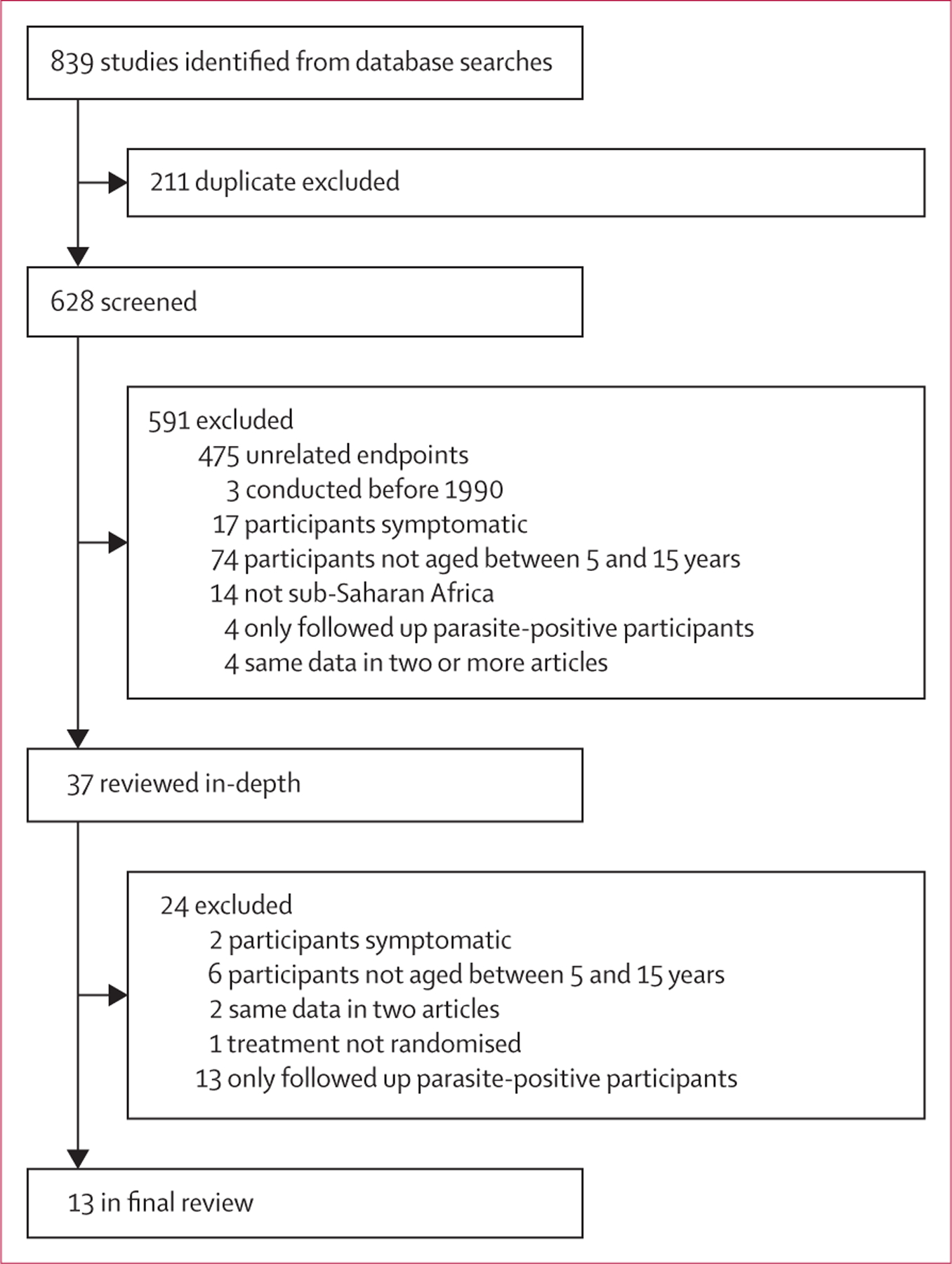
Study selection

**Figure 2:**
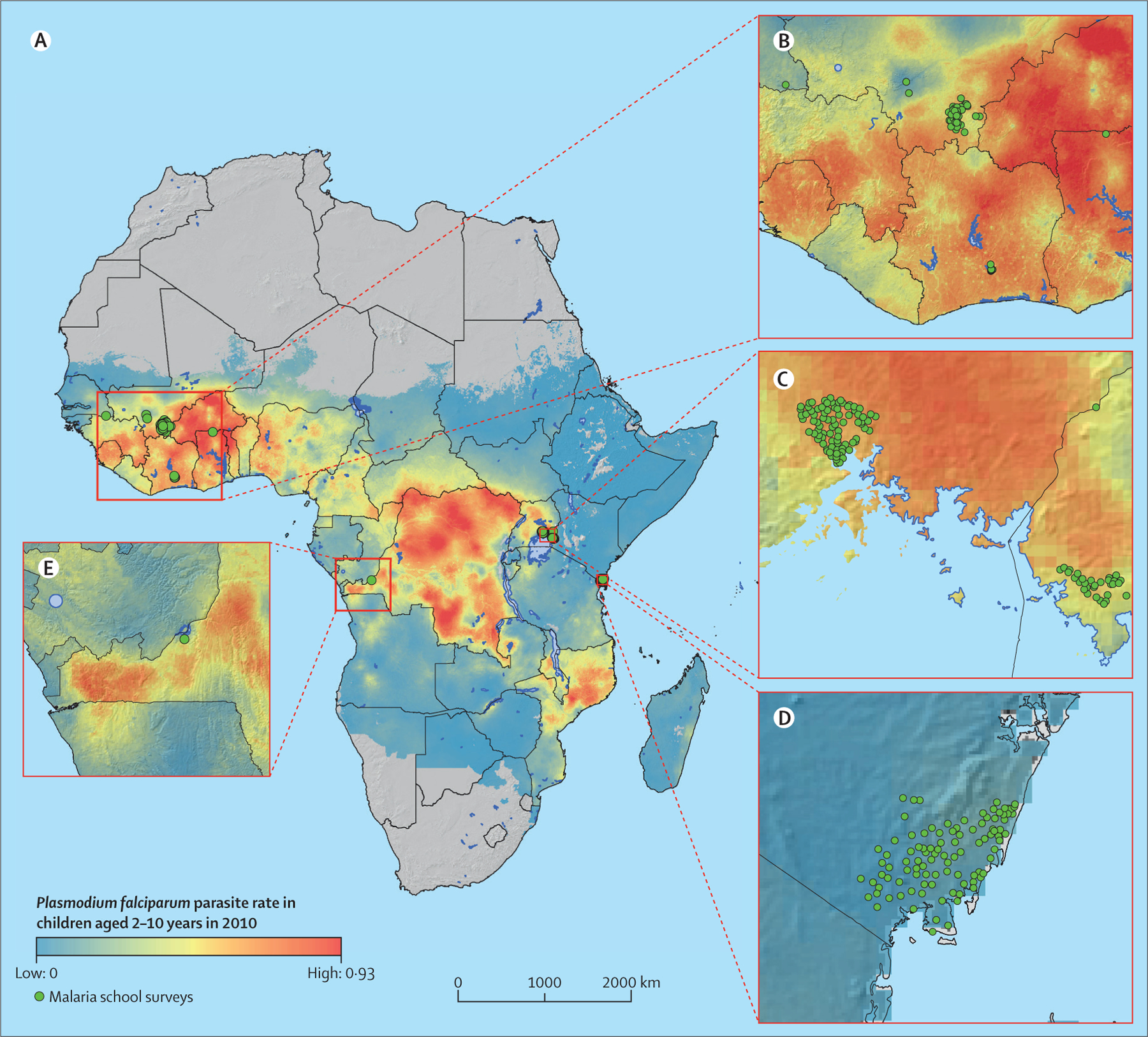
Geographical distribution of included studies (A) in west Africa (B), east Africa (C, D), and central Africa (E) Underlying map shows the predicted *Plasmodium falciparum* parasite rate among children aged 2–10 years in 2010 (Malaria Atlas Project).^31^

Of 628 studies screened, 13 trials met inclusion criteria (figure 1). These trials were done in seven sub-Saharan African countries in locations where malaria prevalence (*Pf*PR_2–10_) ranged from 3% to 67% (figure 2). 11 different drug combinations were used with dosing that ranged from a single treatment course to monthly treatment for 6 months (table 1). Nine trials were individually randomised; four were cluster randomised. Among 12 of 13 studies, participants in the intervention groups received preventive treatment at enrolment without a malaria diagnosis. This was for the dual purpose of clearing parasites that might have been circulating at that moment and providing malaria chemoprophylaxis. One study, however, only provided treatment to participants who first tested positive for malaria, thereby restricting chemoprophylactic effects to those who tested positive at enrolment.^35^ Collectively, we refer to these as preventive treatment studies. Overall, these studies followed up participants for a median of 43 weeks (range 6–103) and outcomes were measured at a median of 60 days after the last treatment dose (0–180). The median proportion of follow-up time protected by treatment was 49% (2–100%). Summaries of each study are provided in the appendix p 8. Summary data were used in the study-level meta-analysis (n=16 309; 13 trials); research groups provided data for individual participant data meta-analysis (n=15 658; 11 trials; table 2). Of the two trials not included, one research group declined to participate and the second was unable to locate individual-level data.^38,39^

**Figure 3:**
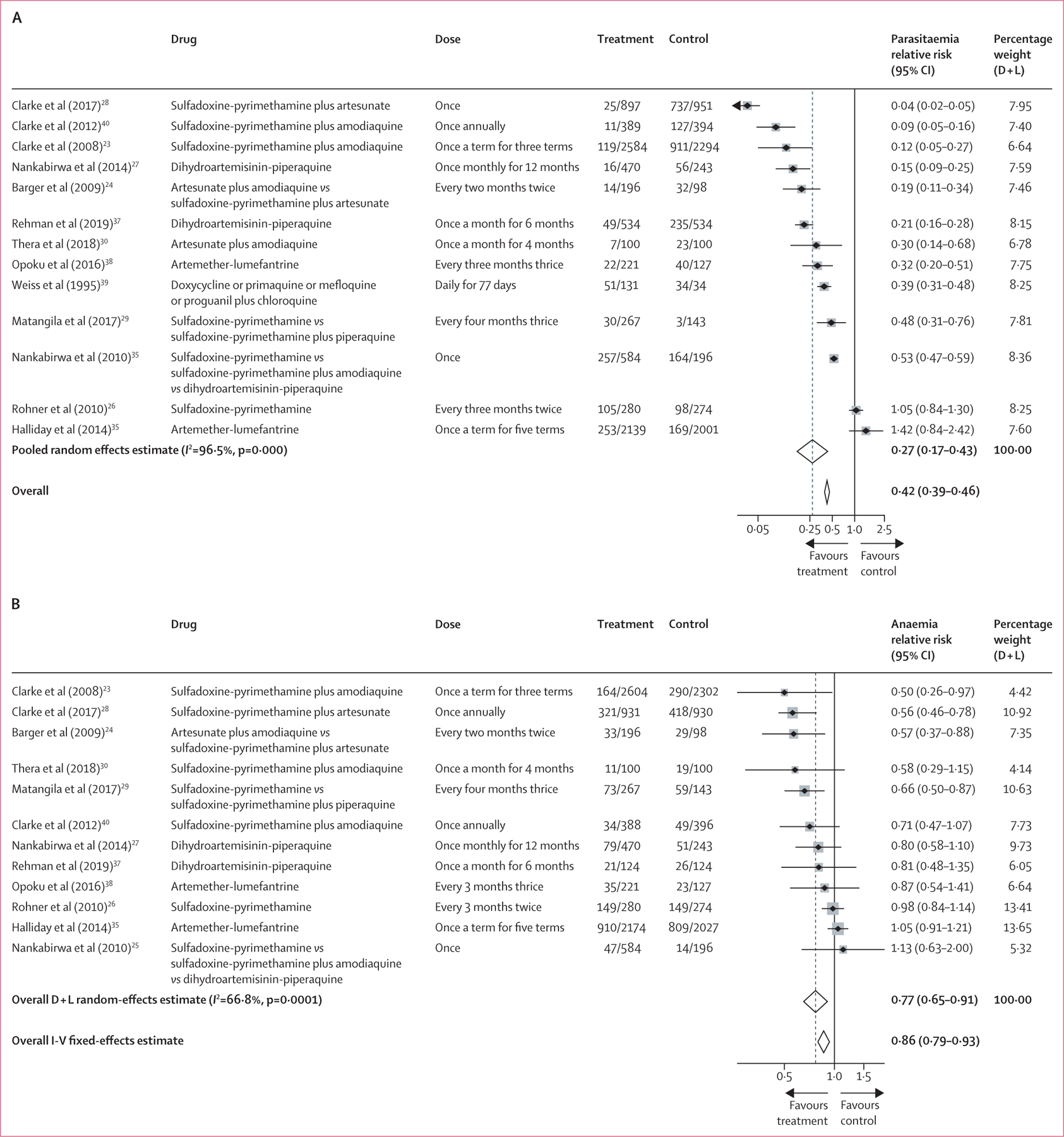
The effect of antimalarial treatment of asymptomatic school-aged children on *Plasmodium falciparum* infection (A) and anaemia (B) D+L=DerSimonian and Laird random effects models. I-V=Inverse variance fixed-effects models. *Pooled random effects estimate. †Pooled fixed effects estimate.

In the study-level meta-analysis, treatment was associated with a 72% reduction in the prevalence of *P falciparum* infection (risk ratio [RR] 0·27, 95% CI 0·17–0·43; figure 3). Among studies in which there was a significant effect, all interventions were beneficial and the range of effect was from 47% to 96% reduction. Only two studies did not show a benefit. One used sulfadoxine–-pyrimethamine alone as the intervention drug,^26^ whereas the other employed a screen-and-treat approach and was done in an area of relatively low prevalence.^35^ In the study-level meta-analysis, treatment was associated with a 23% reduction in anaemia (RR 0·77, 95% CI 0·65–0·91; figure 3). Among studies in which there was a significant effect on anaemia, all interventions were beneficial and the range of effect was from 34% to 50% reduction. There were no clear patterns among studies with or without effect on anaemia with regard to duration of follow-up, proportion of follow-up time protected by treatment, days from last dose of intervention to outcome measurement, or concomitant interventions (eg, anti-helminth treatment). For all outcomes, there was strong evidence of between-study heterogeneity of effect, which meta-regression analyses by drug class, region of study, prevalence of *P falciparum* infection among children aged 2–10 years, proportion of follow-up time protected, or study design did not explain (appendix p 34).

**Figure 4:**
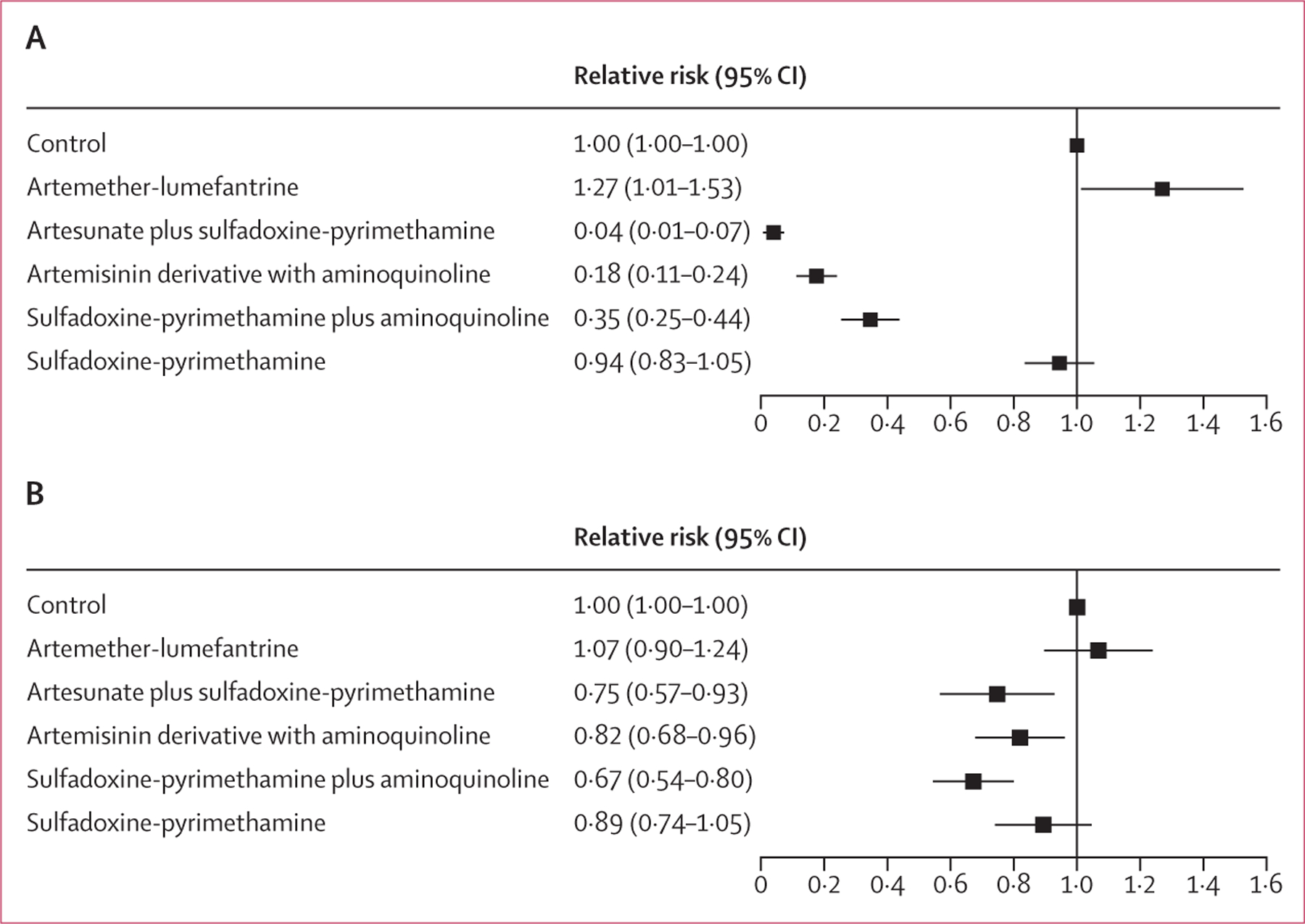
Individual participant data meta-analysis forest plots of the effect of antimalarial treatment by drug type on *Plasmodium falciparum* infection (A) and anaemia (B) across 11 studies with 15 658 individuals Relative risks adjusted for age, sex, and transmission setting.

In the individual participant data meta-analysis, the risk of *P falciparum* infection was approximately halved among participants in intervention groups compared with control groups (adjusted RR [ARR] 0·46, 95% CI 0·40–0·53; p<0·0001; table 3). The reduction in malaria-associated anaemia was 15% (ARR 0·85, 0·77–0·92; p=0·0002). The reduction in risk of *P falciparum* infection and anaemia was related to the treatment regimen (figure 4). Sulfadoxine–pyrimethamine alone was not as effective as when combined with an aminoquinoline (amodiaquine or piperaquine), or when compared with artemisinin-based combination therapy. The one study in the individual participant data meta-analysis that used artemether–lumefantrine did not show protective efficacy. However, this study provided treatment to only children who had infection detected by rapid diagnostic test; therefore, we cannot distinguish between whether it was the screen-and-treat strategy or the drug used that was ineffective. When excluding data from participants who received artemether–lumefantrine or sulfadoxine–pyrimethamine alone, treatment reduced the risk of *P falciparum* infection by 58% (ARR 0·42, 95% CI 0·33–0·50; p<0·0001). As the duration of follow-up time protected by treatment increased, the risk of *P falciparum* infection decreased (table 4). Intervention effect on *P falciparum* infection was similar among children younger than 10 years versus those aged 10–15 years, although there was some evidence of a stronger effect on malaria-related anaemia in younger children (p_interaction_=0·015, appendix p 35).

Treatment was effective in reducing *P falciparum* infection across all transmission settings. The magnitude of effect varied by malaria transmission setting (likelihood ratio test for interaction p<0·0001), but there was no consistent pattern to this interaction (see stratified risk ratios in appendix p 36). This might have been due to the variety of treatment regimens used across the different study sites, but data were too sparse to draw conclusions (appendix p 37). Regardless, the preventive effect of treatment regimens with a higher proportion of follow-up time protected became smaller as the intensity of transmission increased (appendix p 39). There was no evidence of interaction between transmission setting and the effect of treatment on anaemia.

Protection against subsequent clinical malaria was reported in five studies.^24,27,29,30,39^ Treatment reduced the risk of clinical malaria by 60% (RR 0·40, 95% CI 0·28–0·56) in the study-level meta-analysis (appendix p 41). Crude analyses of the four studies in the individual participant data meta-analysis showed a 44% (RR 0·56, 95% CI 0·45–0·67; p<0·0001) reduced risk of clinical malaria and adjusted analyses showed a reduced risk of 50% (ARR 0·50, 95% CI 0·39–0·60; p<0·0001; 1815 individuals; four studies; table 3). The drug combinations used were all effective with the exception of sulfadoxine–pyrimethamine alone (appendix p 42). Although reduction in the risk of clinical malaria was similar in studies with intermediate and high proportion of follow-up time protected by treatment (table 4), treatment with dihydroartemisinin–piperaquine once a school term in an area of high transmission did not significantly reduce clinical malaria; however, treatment monthly during the school year was effective.^27^ There was no difference in the effect by age-group (appendix p 36), nor was there interaction between transmission setting and the effect of treatment on clinical malaria.

Treatment effect on cognitive outcomes was investigated most commonly using the code transmission test, applied in six trials overall, of which five contributed individual participant data.^23,27,28,35,38,40^ Study-level results are not presented because only three (50%) of six trials were published with results presented in a way that was amenable to analysis, and pooled estimates from individual participant data did not show an improvement following treatment (adjusted mean difference 0·12, 95% CI –0·20 to 0·43; p=0·456; table 3). However, when data were stratified by age, there was evidence of a difference in intervention effect on test scores by age group (p_interaction_=0·004), with a modest increase in test scores among children aged 10–15 years (+0·36, 95% CI 0·01 to 0·71; p=0·044; appendix p 35).

Overall risk of bias was low. Performance bias was most common as participants and personnel giving the treatments were usually not masked. Even in the four placebo-controlled trials, the tablets used differed in taste, increasing the possibility for allocation to become unmasked. However, detection bias was relatively low as investigators assessing outcomes were blinded in most studies (appendix p 8; appendix p 42). Funnel plots showed none of the patterns associated with reporting bias, but were consistent with the observed heterogeneity in between-study estimates of effect (appendix p 43).

Two of the trials that contributed the most data in the participant-level meta-analysis also differed from the other studies, either by study design or implementation (table 1). Halliday and colleagues^35^ screened for *P falciparum* infection and provided treatment only to positive cases, whereas in all other studies children received treatment without parasite status known. Treatment coverage levels were 40% or higher in all trials except Staedke and colleagues^36^ in which less than 10% of participating children received the maximum number of treatment rounds, due to challenges with recruitment and absenteeism. Sensitivity analysis excluding these data did not change the significance of associations, but it did increase the estimated effect size (appendix p 38).

11 studies reported adverse events. No deaths were attributed to study drugs. Three studies found differences between intervention and control groups, which were generally symptoms such as dizziness, nausea, and vomiting shortly after treatment.^23,25,29^ Details are provided in the study summaries (appendix p 8).

## Discussion

These two meta-analyses provide strong and consistent evidence that preventive malaria treatment among school-aged children decreases *P falciparum* infection, clinical malaria, and malaria-related anaemia. Importantly, our results suggest that school-based interventions benefit children across all levels of malaria transmission, including geographic areas with very low (3%) to very high (67%) parasite prevalence. Combination drug regimens and increasing the duration of time protected by treatment improved the protection conferred by preventive treatment. While fewer studies measured the impact of treatment on clinical malaria, those that did showed a substantial benefit. The 50% reduction in clinical malaria episodes is similar to the reduction observed in early studies of bednets treated with insecticide—a widely implemented malaria control measure—as well as seasonal malaria chemoprevention in children younger than 5 years, which is now standard in the Sahel.^22,41^

Preventive treatment was also associated with a statistically significant reduction in anaemia, although the magnitude of the reduction was greater in study-level analysis (23%) compared with individual participant data analysis of all data (15%) and sensitivity analysis (21%). These differences might be due to the included studies and the analytic methods applied.^42^ One trial included in the study-level meta-analyses did not provide data for the individual participant data meta-analysis, and there were differences in how the effects from cluster randomised controlled trials were included in the two meta-analytic approaches: cluster-adjusted effects if available, or raw counts scaled by design effect due to clustering, were used in the summary data meta-analysis, whereas a random effect for clusters (in addition to one for studies) was used in the individual participant data meta-analysis. Although this effect appears relatively modest, even daily or weekly iron supplementation reduces anaemia in this age group by only 50%.^43,44^ In Mali, a one-time antimalarial treatment was associated with a larger reduction in the prevalence of anaemia than were weekly-doses of iron supplementation given for 10 weeks in a previous study in the same area.^28,45^ Anaemia in most malaria-endemic areas is attributable to multiple factors including malaria, helminth infections, chronic inflammation, chronic undernutrition, and micronutrient deficiencies.^46^ School-based antimalarial treatment addresses only the fraction of anaemia attributable to malaria, which might explain why most treatment studies had a dramatic effect on *P falciparum* infection, but less effect on anaemia. Thus, our meta-analysis might underestimate the benefit of preventive treatment on anaemia.

Similarly, the absence of improvement in cognitive test scores after treatment might be explained by the complexity of influences on cognitive function in this age group and the duration of interventions. While decreases in cognitive function have been linked to both cerebral malaria and to asymptomatic parasitaemia,^14^ factors such as poverty, insufficient stimulation at home, poorly resourced schools with large class sizes, poor general health, and inadequate nutrition have all been linked to decreased cognitive function.^47–49^ These factors also interact and contribute differentially to decreased cognitive function and complicate the interpretation of our results. However, several trials reported results of cognitive function improvement after malaria treatment.^23,28^ Our results might differ from these studies due to the exact methods of analysis and the conservative methods we employed to adjust for clustering. Treatment did, however, improve test scores in children aged 10–15 years in stratified analysis. Children aged 10–15 years should have higher proficiency in numeracy and writing, resulting in a better understanding of the test instructions and the ability to record their responses; therefore, results in this age group should be more reliable. For more definitive evidence, additional studies with age-appropriate outcomes sensitive to assessing the effects of preventive treatment on cognitive function in younger school-aged children might be needed.

Our study-level analyses showed that some intervention designs did not do as well as others. Notably, the trial by Halliday and colleagues,^35^ which was the only study to use a screen-and-treat approach, used short-acting artemether–lumefantrine as treatment, that took place in a low transmission setting showed no effect. This study design resulted in participants being protected by treatment for an estimated average of 2% of their follow-up. The inferiority of screen-and-treat interventions, compared with chemoprophylaxis or intermittent preventive treatment, is consistent with interventions to prevent malaria in pregnancy and mass treatment to interrupt transmission.^50,51^ Currently available screening methods do not detect low-density infections, which make up a larger proportion of the infections in low transmission settings.^52^ An additional shortcoming of screen-and-treat approaches is that only children who test positive benefit from the chemoprophylactic effect of treatment against future *P falciparum* infections in the near term.

The antimalarial drugs used in each of these trials are well studied formulations known to be safe and well tolerated. None of the studies reported deaths related to the intervention and no unusual adverse events were reported (appendix p 8). The variety of antimalarial therapies and dosing regimens used among the studies allowed us to explore the effects of different types of drug combinations, as well as the proportion of time protected. Not surprisingly, sulfadoxine–pyrimethamine alone was less effective than artemisinin-based combination therapies or sulfadoxine–pyrimethamine combined with an aminoquinoline due to widespread resistance, particularly in east Africa. We could not factor in levels of resistance to sulfadoxine–pyrimethamine, which could limit both the effectiveness of treatment and the duration of chemoprophylaxis after treatment. Therefore, we might have overestimated the protected time in studies using sulfadoxine–pyrimethamine alone or in combination with shorter acting drugs. Although there were differences in the size of the effect of treatment on *P falciparum* infection by transmission setting, the key finding is that treatment was effective in all regions and transmission settings.

Although these analyses focused on the effect of treating asymptomatic school children at the individual level, school-based malaria treatment can confer an additional community-level effect by decreasing local transmission. School-aged children are significant reservoirs of human-to-mosquito transmission.^16,17^ Three studies,^27,28,37^ reported that intervention significantly decreased the prevalence of gametocytes, the parasite life-stage required for transmission. Staedke and colleagues^36^ found that treating school-aged children decreased infection in the surrounding community by a small but statistically significant proportion. This, despite low coverage, was consistent with seasonal malaria chemoprevention among school-age children where other age groups not target for intervention have experienced concomitant reductions in parasitaemia.^53^

The primary limitation to these analyses is the variability between studies, including differences in the intensity and seasonality of malaria transmission in study sites, differences in the intervention drugs and frequency of dosing, as well as differences in the timing of measuring outcomes. A random-effects regression model of individual participant data with adjustment for study and participant characteristics was used to account for some sources of heterogeneity and incorporate any residual heterogeneity into the pooled effect estimates. Ultimately, this variability limits our ability to define the optimal intervention strategy for each setting. Based on our results, we can suggest guiding principles for initial policy and programmatic interventions, as well as studies to further optimise interventions.

Our results are robust and provide evidence for development of policy and programmatic interventions. School-based preventive treatment is effective across a wide range of transmission settings in sub-Saharan Africa. In areas with higher parasite prevalence and thus more intense transmission, higher proportions of time protected are required, either by using drugs with longer half-lives above therapeutic efficacy levels, or by more frequent dosing of shorter-acting drugs. In highly seasonal settings a single treatment at the end of the transmission season provides substantial benefits,^28,40^ whereas in areas with year-round transmission, treatment each school term would be most practical. However, monthly treatment might be required in areas with high perennial transmission.^27^ In the studies included in these analyses, artemisinin-based combination therapies or sulfadoxine–pyrimethamine with an aminoquinoline were effective drug combinations. However, because large scale chemoprevention efforts might increase drug pressure and resistance, the local first-line treatment should not be used for school-based treatment.^51^ Therefore, artesunate–amodiaquine and artemether–lumefantrine for preventive approaches should be avoided in some settings. Dihydroartemisinin–piperaquine is favourable as it is frequently used as first-line treatment in Africa, has a long-half life, and has been used effectively in multiple studies. However, the only study to measure directly the effect of school-based treatment on drug resistance showed that recent treatment with dihydroartemisinin–piperaquine was associated with higher prevalence of molecular markers of drug resistance.^54^ Sulfadoxine–pyrimethamine in combination with another compound should be weighed carefully given changing resistance patterns. The ideal drug characteristics for this purpose include combination therapies with well matched half-lives, drugs with competing resistance mechanisms from first-line treatment drugs, or a rotation of drugs. The benefits of preventive treatment in this population must be weighed against the potential risk of drug resistance.

School-based preventive treatment should be considered for implementation alongside vector control and other interventions to increase protective effects, reduce community transmission, and limit opportunities to select for resistant parasites.^51,55^ Vector control has been widely applied and benefits the total population. Yet, the prevalence of infection in school-aged children remains high and additional interventions are needed to target this population. School-based preventive treatment merits further consideration as an addition to standard malaria control interventions in these areas.

Providing preventive treatment to school-aged children could theoretically hinder the acquisition or maintenance of immunity. This, however, has not been widely observed in studies of intermittent preventive treatment among infants or chemoprevention in children.^56–58^ Importantly, as transmission declines in malaria-endemic areas, evidence suggests that the prevalence of *P falciparum* infection and malaria disease will likely increase in school-aged children.^59–61^ Thus, developing interventions to target this age group will help to counter this epidemiological shift in infection and disease burden.^9^ Additionally, access to primary school and school attendance rates are increasing in sub-Saharan Africa, providing an efficient delivery point for preventive treatment similar to school-based deworming campaigns and nutrition programmes.^62,63^ Indeed, providing preventive treatment alongside other interventions could yield synergistic effects to decrease anaemia, improve cognitive function, and educational attainment. Initial policy support for this intervention would facilitate operational and implementation research to evaluate alternative drugs or drug strategies, assess the effect of combined interventions, investigate the community-level effect of school-based treatment on transmission, monitor for rebound morbidity and mortality, and determine cost-effectiveness under operational conditions. Moreover, these results would enable policy makers to weigh up the risks and benefits of the intervention.

Despite historic strides towards malaria elimination over the past 15 years, progress hangs in the balance. Additional interventions are urgently needed, particularly ones that target populations responsible for human-to-mosquito transmission. Our analysis supports preventive treatment of malaria among school-age children that will decrease the burden of disease in this vulnerable age group.

## Supplementary Material

mmc1

## Figures and Tables

**Table 1:** Characteristics of studieçs included in the study-level meta-analysis

	Years	Randomisation level	Outcomes measured	Treatment	Intervention strategy	Treatment interval	Number of treatment courses	Intervention group (n)	Control group (n)	Follow-up time protected by treatment	Coverage‡	*Pf*PR_2–10_^§^
Weiss et al (1995),^39^ Kenya	1993	Individual	Parasitaemia, clinical malaria	Doxycycline *vs*	Chemoprophylaxis	Weekly for mefloquine and chloroquine	11	32	34	79%	NA	NA
				Primaquine *vs*				32	34			
				Mefloquine plus multivitamin *vs*				30	34			
				Proguanil plus chloroquine		Daily for all other interventions	77	37	34			
Clarke et al (2008),^23^ Kenya	2005–06	Cluster	Parasitaemia, anaemia,‡ cognition	Sulfadoxine–pyrimethamine plus amodiaquine	Intermittent preventive treatment	Termly	3	2604	2302	35%	41%	30–40%
Barger et al (2009),^24^ Mali	2007–08	Individual	Parasitaemia, anaemia, clinical malaria	Artesunate plus amodiaquine *vs*	Intermittent preventive treatment	Every 2 months	2	100	98	20%	NA	10%
				Sulfadoxine–pyrimethamine plus artesunate				96	98	58%		
Nankabirwa et al (2010),^25^ Uganda	2008	Individual	Parasitaemia,‡ anaemia	Sulfadoxine–pyrimethamine *vs*	Parasite clearance	Once	1	186	196	83%	NA	36%
				Sulfadoxine–pyrimethamine plus amodiaquine *vs*				200	196	83%		
				Dihydroartemisinin-piperaquine				198	196	70%		
Rohner et al (2010),^26^ Côte d’Ivoire	2006–07	Individual	Parasitaemia, anaemia	Sulfadoxine–pyrimethamine	Intermittent preventive treatment	Every 3 months	2	280	274	29%	NA	48–55%
Clarke et al (2012),^40^ Senegal	2012	Individual	Parasitaemia,§ anaemia,§ cognition§	Sulfadoxine–pyrimethamine plus amodiaquine	Parasite clearance	Once	1	389	396	63%	NA	16%
Halliday et al (2014),^35^ Kenya	2010–12	Cluster	Parasitaemia, anaemia,‡ cognition	Artemether–lumefantrine	Screen and treat	Termly	5	2174	2027	2%	66·8%	3–13%
Nankabirwa et al (2014),^27^ Uganda	2011–12	Individual	Parasitaemia, anaemia, clinical malaria, cognition§	Dihydroartemisinin–piperaquine termly *vs* monthly	Intermittent preventive treatment	Termly	4	234	243	30%	NA	42%
						Monthly	12	36	243	90%		
Opoku et al (2016),^38^ Ghana	2011	Individual	Parasitaemia, anaemia, cognition	Artemether–lumefantrine	Intermittent preventive treatment	Every 3 months	3	221	127	12%	NA	65%
Clarke et al (2017),^28^ Mali	2011–12	Cluster	Parasitaemia, anaemia,‡ cognition	Sulfadoxine–pyrimethamine plus artesunate	Parasite clearance	Once	1	930	931	58%	94·6%	50–67%
Matangila et al (2017),^29^ DRC	2012–13	Individual	Parasitaemia, anaemia, clinical malaria	Sulfadoxine–pyrimethamine alone *vs* with piperaquine	Intermittent preventive treatment	Every 4 months	3	137	143	29%	NA	16%
								130	130	29%		
Staedke et al (2018)^36^ and Rehman et al (2019),^37^ Uganda	2014	Cluster	Parasitaemia,‡ anaemia	Dihydroartemisinin–piperaquine	Intermittent preventive treatment	Monthly	6	546	546	49%	7·1%	7–16%
Thera et al (2018),^30^ Mali	2013–14	Individual	Parasitaemia, anaemia, clinical malaria	Artesunate plus amodiaquine	Intermittent preventive treatment	Monthly	4	100	100	32%	NA	40%

The total number of treatment courses given over the duration of the study. NA=not applicable.

* Coverage for cluster randomised studies; for additional details see appendix.

† *PfPR*_2–10_ is the annual mean prevalence of *Plasmodium falciparum* infection among children aged 2–10 years according to the Malaria Atlas Project.

‡ Primary outcome—numbers in intervention and control groups correspond to the primary outcome if they are not the same for all analyses.

§ Unpublished.

**Table 2:** Characteristics of individuals and study areas in the meta-analyses

	Control (n=7221)	Intervention (n=8437)
Age, years	9·9 (2·7)	10·0 (2·7)
Sex		
Female	3509 (48·7%)	4044 (48·0%)
Male	3695 (51·3%)	4385 (52·0%)
Estimated transmission intensity (*Pf*PR_2–10_) during the trial		
Low (<10%)	1422 (19·7%)	1866 (22·1%)
Low–moderate (10 to <30%)	1959 (27·1%)	2126 (25·2%)
Moderate–high (30 to <50%)	2886 (40·0%)	3597 (42·6%)
High (≥50%)	954 (13·2%)	848 (10·1%)

Data are mean (SD) and n (%). *Pf*PR_2–10_=annual mean prevalence of *Plasmodium falciparum* infection among children aged 2–10 years according to the Malaria Atlas Project.

**Table 3:** Effect of antimalarial treatment on primary and secondary outcomes for the individual participant data meta-analysis

	Control	Intervention	Crude relative risk* (95% CI)	p value	Adjusted relative risk† (95% CI)	p value
*Plasmodium falciparum* infection	2521 (34·9%)	869 (10·3%)	0·50 (0·43 to 0·57)	<0·0001	0·46 (0·40 to 0·53)	<0·0001
Anaemia	1904 (27·9%)	1855 (22·7%)	0·85 (0·78 to 0·93)	0·0002	0·85 (0·77 to 0·92)	0·0002
Clinical malaria during follow-up‡	144 (24·8%)	134 (12·7%)	0·56 (0·45 to 0·67)	<0·0001	0·50 (0·39 to 0·60)	<0·0001
Code transmission test scores§	13·24 (0·10)	13·40 (0·09)	0·15 (–0·17 to 0·46)¶	0·3690	0·12 (–0·20 to 0·43)||	0·4564

Data are n (%) or mean (SE), unless otherwise stated.

* Risk ratios were obtained by marginal standardisation. p values from corresponding logistic regression.

† Adjusted for age, sex, and transmission intensity.

‡ Four studies contributing 637 individuals in the control group and 1178 in the intervention group.

§ Five studies contributing 2840 individuals in the control group and 3226 in the intervention group.

¶ Crude difference (95% CI).

|| Adjusted difference (95% CI).

**Table 4:** Effects of low, intermediate, and high proportion of follow-up time protected by treatment on outcomes

	Control	Proportion follow-up time protected by treatment			Adjusted relative risk*		
	
		Low	Intermediate	High	Low protected time (95% CI)	Intermediate protected time (95% CI)	High protected time (95% CI)
*Plasmodium falciparum* infection	2521 (34·9%)	253 (11·8%)	314 (7·4%)	302 (14·8%)	1·28 (0·99 to 1·56)	0·60 (0·50 to 0·70)	0·24 (0·15 to 0·32)
Anaemia	1904 (27·9%)	910 (41·9%)	510 (13·3%)	435 (20·8%)	1·07 (0·90 to 1·24)	0·79 (0·69 to 0·90)	0·77 (0·64 to 0·90)
Clinical malaria during follow-up†	144 (24·8%)	··	88 (10·2%)	46 (23·5%)	··	0·54 (0·40 to 0·68)	0·42 (0·25 to 0·59)
Educational test scores‡	13·18 (0·10)	13·93 (0·11)	13·67 (0·16)	9·10 (0·27)	–0·09 (–0·53 to 0·35)§	0·48 (–0·06 to 1·01)§	0·03 (–0·75 to 0·82)§

Data are n (%) or mean (SE), unless otherwise stated. Protected time in control group is 0%. Low protected time is less than 20%; intermediate protected time is 20% or more, <50%; high protected time is 50% or more. Adjusted for age, sex, and transmission intensity.

* Relative risks are obtained by marginal standardisation, p values from corresponding logistic regression, and adjusted for age, sex, and transmission intensity.

† Four studies contributing 637 individuals in the control group and 1178 in the intervention group.

‡ Five studies contributing 2840 individuals in the control group and 3226 in the intervention group.

§ Adjusted mean difference.

## Research in context

### Evidence before this study

To our knowledge, Matangila and colleagues, 2015, have published the only previous systematic review of preventive treatment studies among school-aged children, summarising a range of protective efficacy against *Plasmodium falciparum* infection (0·05–94%; four trials), malaria-related anaemia (14–50%; three trials), and clinical malaria during follow-up (0–97%; three trials). No meta-analyses have been done.

### Added value of this study

Our meta-analyses are the first to pool data from preventive antimalarial treatment trials done among asymptomatic schoolchildren. The study-level meta-analysis is based on 13 published and unpublished trials (n=16 309). Our individual-level data meta-analysis draws from 11 trials reporting at least one outcome of interest (n=15 658) to assess the effects of treatment type, duration of protection by treatment regimen, and malaria transmission intensity in each study setting. Our results suggest that preventive antimalarial treatment reduces the prevalence of infection and clinical malaria by 50% and the prevalence of malaria-associated anaemia by 15%. The most effective treatment types were artemisinin-based combination therapies, or sulfadoxine–pyrimethamine combined with an aminoquinoline. Treatments that protected over longer periods of time were more effective, particularly in high transmission settings.

### Implications of all the available evidence

School-aged children in malaria-endemic areas of sub-Saharan Africa have a high prevalence of *P falciparum* infection resulting in malaria-associated anaemia and episodes of clinical malaria, which contribute to poor health and potential under-achievement in school. The school-age population is a major 13 reservoir for human-to-mosquito transmission and is central to perpetuating the cycle of *P falciparum* infection in malaria-endemic areas. Preventive treatment of malaria among school-aged children reduces the burden of malaria in this age group and could be a key intervention to decrease *P falciparum* transmission on the pathway to malaria elimination.
