# Supplementary material for: Preventive malaria treatment among school-aged children in sub-Saharan Africa: a systematic review and meta-analyses
Source: Lancet Glob Health. Author manuscript; Available in PMC 2020 Dec 8. (PMC7721819; doi:10.1016/S2214-109X(20)30325-9)
Supplement: mmc1 [file NIHMS1648016-supplement-mmc1.pdf]

THE LANCET
Global Health

Supplementary appendix

This appendix formed part of the original submission and has been peer reviewed. We post it as supplied by the authors.

Supplement to: Lauren M Cohee LM, Opondo C, Clarke SE, et al. Preventive malaria treatment among school-aged children in sub-Saharan Africa: a systematic review and meta-analyses. *Lancet Glob Health* 2020; published online Oct 22. http://dx.doi.org/10.1016/S2214-109X(20)30325-9.

Supplementary Materials

Supplementary Tables

Table S1. PRISMA Checklist

Table S2. Proportion of follow up time protected by treatment - calculations by study

Table S3. Associations between age and sex and outcomes

Table S4. Study summaries

- **Table S4a.** Weiss *et al* (1995)
- **Table S4b.** Clarke *et al* (2008)
- **Table S4c.** Barger *et al* (2009)
- **Table S4d.** Nankabirwa *et al* (2010)
- **Table S4e.** Rohner *et al* (2010)
- **Table S4f.** Clarke *et al* (2012)
- **Table S4g.** Halliday *et al* (2014)
- **Table S4h.** Nankabirwa *et al* (2014)
- **Table S4i.** Opoku *et al* (2016)
- **Table S4j.** Clarke *et al* (2017)
- **Table S4k.** Matangila *et al* (2017)
- **Table S4l.** Rehman *et al* (2019) and Staedke *et al* (2018)
- **Table S4m.** Thera *et al* (2018)

Table S5. Between-study variance explained by study-level characteristics in meta regression

Table S6. Intervention effects stratified by age-group

Table S7. Effect of treatment on prevalence *Plasmodium falciparum* infection stratified by transmission setting

Table S8. Distribution of overall sample by drug type and malaria transmission setting

Table S9. Sensitivity analysis: Effect of antimalarial intermittent preventative treatment on primary and secondary outcomes excluding Halliday *et al.* 2014 and Staedke *et al.* 2018

Supplementary Figures

Figure S1. Impact of proportion of follow-up time protected by treatment on *Plasmodium falciparum* infection by transmission setting

Figure S2. Forest plot of treatment on clinical malaria by study: Study-level meta-analysis fixed and random effects analyses

Figure S3. Forest plot of clinical malaria by study drug: Individual participant meta-analysis

Figure S4. Forest plot of code transmission test by study drug: Individual participant meta-analysis

Figure S5. Risk of bias within studies

Figure S6. Risk of bias across studies

Figure S7. Funnel plot for the random-effects analysis of *Plasmodium falciparum* infection

Figure S8. Funnel plot for the random-effects analysis of anaemia

Figure S9. Funnel plot for the random-effects analysis of clinical malaria

Supplementary Text

Text S1. Search strategy details

1. PubMed (pubmed.com)
2. Embase (embase.com)
3. Cochrane Central Register of Controlled Trials (Wiley)
4. Clinicaltrials.gov

Text S2. Creation of variable for proportion of follow-up time protected

Text S3. Additional details on coverage in cluster randomized studies

Table S1. PRISMA Checklist

Section/topic	#	Checklist item	Reported on page #
**TITLE**			
Title	1	Identify the report as a systematic review, meta-analysis, or both.	1
**ABSTRACT**			
Structured summary	2	Provide a structured summary including, as applicable: background; objectives; data sources; study eligibility criteria, participants, and interventions; study appraisal and synthesis methods; results; limitations; conclusions and implications of key findings; systematic review registration number.	2-3
**INTRODUCTION**			
Rationale	3	Describe the rationale for the review in the context of what is already known.	5
Objectives	4	Provide an explicit statement of questions being addressed with reference to participants, interventions, comparisons, outcomes, and study design (PICOS).	5
**METHODS**			
Protocol and registration	5	Indicate if a review protocol exists, if and where it can be accessed (e.g., Web address), and, if available, provide registration information including registration number.	6, Supp p.36
Eligibility criteria	6	Specify study characteristics (e.g., PICOS, length of follow-up) and report characteristics (e.g., years considered, language, publication status) used as criteria for eligibility, giving rationale.	6
Information sources	7	Describe all information sources (e.g., databases with dates of coverage, contact with study authors to identify additional studies) in the search and date last searched.	6
Search	8	Present full electronic search strategy for at least one database, including any limits used, such that it could be repeated.	Supp p.36
Study selection	9	State the process for selecting studies (i.e., screening, eligibility, included in systematic review, and, if applicable, included in the meta-analysis).	6
Data collection process	10	Describe method of data extraction from reports (e.g., piloted forms, independently, in duplicate) and any processes for obtaining and confirming data from investigators.	6
Data items	11	List and define all variables for which data were sought (e.g., PICOS, funding sources) and any assumptions and simplifications made.	6
Risk of bias in individual studies	12	Describe methods used for assessing risk of bias of individual studies (including specification of whether this was done at the study or outcome level), and how this information is to be used in any data synthesis.	6
Summary measures	13	State the principal summary measures (e.g., risk ratio, difference in means).	7-8

Section/topic	#	Checklist item	Reported on page #
Synthesis of results	14	Describe the methods of handling data and combining results of studies, if done, including measures of consistency (e.g., I^2) for each meta-analysis.	7-8
Risk of bias across studies	15	Specify any assessment of risk of bias that may affect the cumulative evidence (e.g., publication bias, selective reporting).	6, Supp p. 8-27, 30-33, 35
Additional analyses	16	Describe methods of additional analyses (e.g., sensitivity or subgroup analyses, meta-regression), if done, indicating which were pre-specified.	8
**RESULTS**			
Study selection	17	Give numbers of studies screened, assessed for eligibility, and included in the review, with reasons for exclusions at each stage, ideally with a flow diagram.	9, Figure 1
Study characteristics	18	For each study, present characteristics for data extracted (e.g., study size, PICOS, follow-up period) and provide the citations.	9 Table 1, Table 2
Risk of bias within studies	19	Present data on risk of bias of each study and, if available, any outcome level assessment (see item 12).	12, Supp p. 8-27, 35
Results of individual studies	20	For all outcomes considered (benefits or harms), present, for each study: (a) simple summary data for each intervention group (b) effect estimates and confidence intervals, ideally with a forest plot.	10-12, Figure 3, Supp p. 33
Synthesis of results	21	Present results of each meta-analysis done, including confidence intervals and measures of consistency.	10-12, Figure 3, Figure 4, Table 4, Table 5 Supp p. 34
Risk of bias across studies	22	Present results of any assessment of risk of bias across studies (see Item 15).	12, Supp p. 30-33
Additional analysis	23	Give results of additional analyses, if done (e.g., sensitivity or subgroup analyses, meta-regression [see Item 16]).	10-12. Supp p. 28-29, Table 3
**DISCUSSION**			

Summary of evidence	24	Summarize the main findings including the strength of evidence for each main outcome; consider their relevance to key groups (e.g., healthcare providers, users, and policy makers).	13-15
Limitations	25	Discuss limitations at study and outcome level (e.g., risk of bias), and at review-level (e.g., incomplete retrieval of identified research, reporting bias).	13-16
Conclusions	26	Provide a general interpretation of the results in the context of other evidence, and implications for future research.	16-18
**FUNDING**			
Funding	27	Describe sources of funding for the systematic review and other support (e.g., supply of data); role of funders for the systematic review.	9

Table S2. Proportion of follow up time protected by treatment - calculations by study (See text S2 for details on the calculations presented here)

Study	Drug	Treatment Interval	Treatment frequency	Post-treatment prophylaxis (days)	Days protected by treatment	Days between first dose and outcome measurement	Proportion follow up period protected by treatment
Weiss et al (1995)	Doxy	daily	77	Continuous chemoprophylaxis	77	77	100%
	PQ	daily	77		77	77	100%
	MQ (MVI)	weekly (daily)	11		77	77	100%
	Proguanil+CQ	daily - weekly	77 - 11		77	77	100%
Clarke et al (2008)	SP+AQ	four-monthly	3	35	105	300	35%
Barger et al (2009)	AS+AQ	two-monthly	2	14.4	24	120	20%
	SP+AS	two-monthly	2	35	70	120	58%
Nankabirwa et al (2010)	SP	once	1	35	35	42	83%
	SP+AQ	once	1	35	35	42	83%
	DP	once	1	29.4	29.4	42	70%
Rohner et al (2010)	SP	three-monthly	2	35	70	240	29%
Halliday et al (2014)	AL	termly	5	13.8	12.075*	720	2%
Nankabirwa et al (2014)	DP	termly	4	29.4	117.6	390	30%
	DP	monthly	12	29.4	352.8	390	90%
Opoku et al (2016)	AL	three-monthly	3	13.8	41.4	360	12%
Clarke et al (2017)	SP+AS	once	1	35	35	60	58%
Matangila et al (2017)	SP	four-monthly	3	35	105	360	29%
	SP+PQ	four-monthly	3	35	105	360	29%
Staedke et al (2018)	DP	monthly	6	29.4	176.4	360	49%
Thera et al (2018)	SP+AQ	monthly	4	14.4	57.6	180	32%
Clarke et al (2012)	SP+AQ	once	1	35	35	56	63%

*Screen-and-treat design – an average of 17.5% of children in the intervention arm were positive based on RDT results

Table S3. Associations between age and sex and outcomes

Outcome	Age in years (95%CI), p-value				Sex (males relative to females) risk ratios (95%CI), p-value			
	**Unadjusted RR**		**Adjusted RR**		**Unadjusted RR**		**Adjusted RR**	
Plasmodium falciparum infection†	0.96 (0.96 – 0.97)	p < 0.001	0.96 (0.95 – 0.97)	p < 0.001	1.06 (1.01 – 1.10)	p = 0.006	1.08 (1.03 – 1.13)	p = 0.002
Anaemia†	0.99 (0.98 – 1.00)	p = 0.054	0.99 (0.98 – 1.00)	p = 0.049	1.08 (1.02 – 1.14)	p = 0.004	1.09 (1.03 – 1.15)	p = 0.003
Clinical malaria during follow-up‡	0.93 (0.91 – 0.95)	p < 0.001)	0.91 (0.90 – 0.92)	p < 0.001	0.89 (0.73 – 1.04)	p = 0.175	0.84 (0.67 – 1.02)	p = 0.109
	**Unadjusted difference**		**Adjusted difference**		**Unadjusted difference**		**Adjusted difference**	
Code transmission test scores§	0.26 (0.22 – 0.31)	p < 0.001	0.26 (0.22 – 0.31)	p < 0.001	0.07 (-0.15 – 0.29)	p = 0.548	0.02 (-0.20 – 0.24)	p = 0.845

RR = Risk ratios that were obtained by marginal standardization; p-values from corresponding logistic regression and adjusted for sex or age, as relevant, treatment group and transmission intensity

† Eleven studies contributing 15,658 observations

‡ Four studies contributing 1,815 observations

§ Five studies contributing 6,066 observations

Table S4. Study Summaries

Table S4a. Weiss *et al* (1995)

**Methods**	**Trial design**: Double-blind, individually-randomized, 4-arm trial of chemoprophylaxis<br><br>**Follow-up**: Daily doses were administered at school by a study field worker, participants were visited daily at home to assess symptoms of malaria or drug side effects and obtain weekly blood smears<br><br>**Adverse event monitoring**: Daily visits by field worker, blood chemistries at 6 and 11 weeks
**Participants**	**Number of participants randomised**: 169<br><br>**Inclusion criteria**: 9- to 14-year-olds; Normal physical exam; Normal complete blood count, blood urea nitrogen, serum alanine aminotransferase, glucose-6 phosphate dehydrogenase levels, haemoglobin electrophoresis<br><br>**Exclusion criteria**: Positive urine pregnancy test
**Interventions**	**Arm 1**: Doxycycline 50mg, daily for 11 weeks<br><br>**Arm 2**: Primaquine 15mg base, daily for 11 weeks<br><br>**Arm 3**: Mefloquine 125mg, weekly + multivitamin, daily for 11 weeks<br><br>**Arm 4**: Proguanil 200mg, daily + chloroquine 150mg, daily for 11 weeks<br><br>**Control**: Multivitamin, daily for 11 weeks
**Outcomes**	**Outcomes included in the review**<br><br>• Parasitaemia (smear)<br>• Clinical malaria (symptoms + positive smear)<br><br>**Time (days) from last dose to outcome measurement**: 0, outcomes were measured intermittently through the intervention and presented as cumulative percentages at the end of treatment
**Notes**	**Country**: Kenya<br><br>**Setting**: 4 primary schools<br><br>***Pf*PR$_{2-10}$**: Not available<br><br>**Coverage**: Not applicable as individually randomized study<br><br>**Estimated proportion of time protected**: 100%<br><br>**Other interventions**: all participants were treated at baseline with quinine<br><br>**Adverse events**: No significant differences<br><br>**Drug resistance data**: None<br><br>**Funding**: Not specified

Risk of bias		
**Bias**	**Authors' judgement**	**Support for judgement**
Random sequence generation (selection bias)	Unclear	Randomized within each school, but details of randomization were not provided.
Allocation sequence (selection bias)	Unclear	Details of allocation concealment were not provided.
Blinding (performance bias and detection bias) All outcomes	Unclear	"Drugs provided in brown envelopes and were administered ... by one field worker at each school."
Blinding of outcome assessment (detection bias) All outcomes	Low	"None of the malaria slide readers knew which drugs the subjects were taking. None of the field workers visiting the homes daily to ask about symptoms or clinical staff evaluating and treating subjects at the Saradidi Clinic knew which drugs the subjects were taking."
Incomplete outcome data (attrition bias) All outcomes	Low	For the intermittent study 12/91 (13%) did not have complete follow up, 7/169 (4%) did not have complete follow up. Loss to follow-up well explained.
Selective reporting (reporting bias)	Unclear	Without a trial protocol it is unclear whether additional outcomes were measured but not reported based on the results.
Other bias	Low	No other sources of bias were identified.

Table S4b. Clarke *et al* (2008)

Methods	**Trial design**: Double-blind, cluster-randomised, placebo-controlled trial of intermittent preventive treatment<br><br>**Follow-up**: Post-intervention cross-sectional survey approximately 6 weeks after the final dose<br><br>**Adverse event monitoring**: Active monitoring for three days after each treatment, Passive monitoring for 28 days by school staff and local health centre with transport reimbursement
Participants	**Number of participants randomised**: 6,758<br><br>**Inclusion criteria**: Parental consent available; Attendance in a sampled school<br><br>**Exclusion criteria**: History of adverse reaction to sulfa-based drugs; Baseline haemoglobin concentration <70g/L; Known or suspected homozygous sickle-cell trait; Known of suspected pregnancy (not tested)
Interventions	**Arm 1**: Sulfadoxine-pyrimethamine one dose + Amodiaquine three daily doses, administered each term (approx. every 4 months), three times (dosing by age)<br><br>**Control**: Dual placebo tabs over three consecutive days, administered each term (every 4 months), three times
Outcomes	**Outcomes included in the review**<br><br>• Anaemia (Hb<110g/L by HemoCue)<br>• Parasitaemia (smear)<br>• Cognition [tests of sustained attention (code transmission and counting sounds in a cohort of 11- to 16-year-olds), behavioural assessment, educational attainment)<br><br>**Time (days) from last dose to outcome measurement**: approximately 42
Notes	**Country**: Kenya<br><br>**Setting**: 30 primary schools<br><br>***Pf*PR$_{2-10}$**: 30-40% (measured *Pf* prevalence in control group at baseline 37%)<br><br>**Coverage**: 41% (3 days of treatment, on all 3 treatment rounds)<br><br>**Estimated proportion of time protected**: 35%<br><br>**Other interventions**: Children in all schools (intervention and control) received twice yearly deworming with albendazole. Children with Hb<70g/L were given haematinics, *Schistosoma mansoni* positive children were treated with praziquantel. Adverse events: "Seven children died during the 12-month intervention period: two in the IPT intervention group and five in the placebo group. Cause of death was investigated, but could not be reliably ascertained in all cases. 23 serious adverse events were reported within 28 days of any treatment (19 in the IPT group and four in the placebo group); the main side-effects were problems of balance, dizziness, feeling faint, nausea, and/or vomiting shortly after treatment."<br><br>**Drug resistance data**: None

	**Funding**: Bill and Melinda Gates Foundation through Gates Malaria Partnership, Norwegian Education Trust Fund and multi-donor Education Development Programme Fund (World Bank), DBL Centre for Health Research and Development, and Wellcome Trust	
Risk of bias		
**Bias**	**Authors' judgement**	**Support for judgement**
Random sequence generation (selection bias)	Low	Within school performance strata "…schools were randomly allocated to one of six coded drug groups by use of block randomization according to a computer-generated random number list by an investigator blind to the drug group."
Allocation sequence (selection bias)	Low	Drugs were coded and code held only by DSM and drug manufacturer
Blinding (performance bias and detection bias)<br>All outcomes	High	Placebo-controlled trial, study authors noted that, "Active drugs and placebos were similar in size and shape, but differed in taste."
Blinding of outcome assessment (detection bias)<br>All outcomes	Low	"Staff responsible for measuring health and education outcomes were unaware of drug group allocation, and analysis was done by a statistician with no previous involvement in the trial."
Incomplete outcome data (attrition bias)<br>All outcomes	Low	73% of children enrolled were examined in the post-intervention survey 12 months later. Numbers of children lost to follow-up was similar in intervention and control groups, 26% and 28%.
Selective reporting (reporting bias)	Low	Reported primary and secondary outcomes of interest to this review are the same as reported on clinicaltrials.gov.
Other bias	Low	No other sources of bias were identified.

Table S4c. Barger *et al* (2009)

Methods	**Trial design**: Open-label, individually-randomised, placebo-controlled 2-arm trial of intermittent preventive treatment<br><br>**Follow-up**: Participant were followed monthly with history, physical exam, and blood smear. Primary endpoint was two months after the second treatment dose. Passive surveillance for clinical malaria was conducted. A final follow-up visit seven months after the last dose was added to the study.<br><br>**Adverse event monitoring**: Not described
Participants	**Number of participants randomised**: 296<br><br>**Inclusion criteria**: Matriculation in the village school; 6- to 13-year-olds; Absence of severe acute illness; Ability to attend follow-up visits; Written and expressed informed consent/assent from student, parent and teacher<br><br>**Exclusion criteria**: History of allergy to study medications; History of chronic disease
Interventions	**Arm 1**: Artesunate 4mg/kg daily for three days + Amodiaquine 10mg/kg daily for three days, administered every two months, two times<br><br>**Arm 2**: Sulfadoxine-pyrimethamine 25/1.25 mg/kg once + Artesunate 4mg/kg daily for three days, administered every two months, two times<br><br>**Control**: Vitamin C 250mg daily for three days, every two months, two times
Outcomes	**Outcomes included in the review**<br><br>• Parasitaemia (smear)<br>• Anaemia (Hb<11g/dL)<br>• Clinical malaria (symptoms + positive blood smear)<br><br>**Time (days) from last dose to outcome measurement**: approximately 60 days
Notes	**Country**: Mali<br><br>**Setting**: Single school<br><br>***Pf*PR$_{2-10}$**: 10% (measured *Pf* prevalence in control group at baseline 7%)<br><br>**Coverage**: Not applicable as individually randomized study<br><br>**Estimated proportion of time protected**: 20% (AS+AQ); 58% (SP-AQ)<br><br>**Other interventions**: None<br><br>**Adverse events**: "The rate of headache was 8.3% and 4.2% for SP/AS, 13% and 14% for AQ/AS and 10.2% and 7.1% for vitamin C for the first and second treatment courses, respectively. The second most common complaint was abdominal pain with rates of 7.3% and 1.0% for SP/AS, 2.0% and 4.0% for AQ/AS and 0.0% and 3.1% for vitamin C for the first and second courses, respectively. … No students elected to quit the study because of these events." No severe adverse events were reported.<br><br>**Drug resistance data**: Cites unpublished data that both arms had efficacy of >95%

	**Funding**: University of Bamako, Fogarty International Center, European and Developing Countries Clinical Trial Partnership, Howard Hughes Medical Institution, National Institutes of Health	
Risk of bias		
**Bias**	**Authors' judgement**	**Support for judgement**
Random sequence generation (selection bias)	Low	"Students were assigned a computer-generated random number which linked them to one of the three study arms."
Allocation sequence (selection bias)	Low	"As children presented for enrollment, they were assigned a study number in numerical order of presentation within a given grade" No specific description of concealment of allocation.
Blinding (performance bias and detection bias)<br>All outcomes	High	"Researchers were aware at the time of initial treatment dose the arm to which each student was assigned." No comment on participant blinding, but placebo was not used and dosing strategies were different between the arms.
Blinding of outcome assessment (detection bias)<br>All outcomes	High	Per correspondence with authors. Assessment of parasitemia was blinded but assessments of hemoglobin and clinical malaria were not.
Incomplete outcome data (attrition bias)<br>All outcomes	Low	"…total loss to follow-up of 2 (0.7%) students at the end of the initial trial period...additional 10 students lost to follow-up at the study visit added in May 2008."
Selective reporting (reporting bias)	Low	Per correspondence with authors, all planned outcomes were reported.
Other bias	Low	No other sources of bias were identified.

Table S4d. Nankabirwa *et al* (2010)

Methods	**Trial design**: Double-blind, individually-randomized, placebo-controlled trial of parasite clearance<br><br>**Follow-up**: Participants were followed at school with history and physical exam on days 1, 2, 3, 7, 14, 28, 42, and any additional day that they felt ill. Children absent were visited at home. Fingerprick blood samples for smears and filter papers were taken on days 7, 14, 28, 42, (and on any unscheduled day that fever was reported). Haemoglobin was reassessed on day 42.<br><br>**Adverse event monitoring:** During follow-up and structured questionnaire on day 7
Participants	**Number of participants randomised**: 794<br><br>**Inclusion criteria**: Girls 8- to 12-years-old, boys 8- to 14-years-old; Enrolled in classes 1-7; Consent and assent provided<br><br>**Exclusion criteria**: Known allergy or prior adverse reaction to study medications; Onset of menstruation; Fever (axillary temperature ≥37.5°C) or history of fever in previous 24 hours; Evidence of severe malaria or danger signs; Ongoing antimalarial treatment; Haemoglobin ≤7.0 g/dL; Parasite density >10,000/microliter
Interventions	**Arm 1**: Sulfadoxine-pyrimethamine 25/1.25 mg/kg once + Placebo on days two and three, administered once<br><br>**Arm 2**: Sulfadoxine-pyrimethamine 25/1.25 mg/kg once + Amodiaquine 10mg/kg daily for three days, administered once<br><br>**Arm 3**: Dihydroartemisinin-piperaquine 6.4/51.2 mg/kg daily for three days, administered once<br><br>**Control**: Placebo administered daily for three days
Outcomes	**Outcomes included in the review**<br><br>• Parasitaemia (smear)<br>• Anaemia (analysed in the study as Hb, for meta-analysis used WHO age/gender specific cut-offs)<br><br>**Time (days) from last dose to outcome measurement**: 42
Notes	**Country**: Uganda<br><br>**Setting**: Two primary schools<br><br>***Pf*PR$_{2-10}$**: 36% (measured *Pf* prevalence in control group at baseline 56%)<br><br>**Coverage**: Not applicable as individually randomized study<br>Estimated proportion of time protected: 83% (SP, SP+AQ); 70% (DP)<br>Other interventions: Children with Hb<10g/dL were treated with ferrous-sulfate x 14d<br>Adverse events: No serious adverse events were reported. The most common adverse events reported were headache, cough, abdominal pain, coryza, skin rash, nausea, vomiting, and diarrhoea. SP+AQ was associated with more adverse events and more vomiting in the first three days compared to placebo. There were no differences in cumulative adverse events between arms by day 42.<br><br>**Drug resistance data**: Study cites previously published research of 28d recrudescence rates of 35% for SP, 18% for SP+AQ, and 0.3% for DP as well as molecular resistance

	marker prevalence of >80% for the quintuple dhps/dhfr mutations, “uncommon” dhfr I164L and dhps A581G mutations, nearly 100% prevalence of pfcrt 76T mutation, and >80% prevalence of pfmdr1 N86Y and D1246Y mutations. None obtained from this study.<br><br>**Funding**: Bill and Melinda Gates Foundation, Wellcome Trust, Doris Duke Charitable Foundation	
Risk of bias		
**Bias**	**Authors' judgement**	**Support for judgement**
Random sequence generation (selection bias)	Low	"Randomization codes were computer generate in blocks of eight by an investigator not directly involved in the project"
Allocation sequence (selection bias)	Low	"codes were...sealed in numbered envelops. The study nurse assigned treatment numbers sequentially"
Blinding (performance bias and detection bias)<br>All outcomes	High	"Children were not informed of their treatment regimen, but the colour and taste of study medications were dissimilar."
Blinding of outcome assessment (detection bias)<br>All outcomes	Low	Study nurse was not blinded, but "all other study personnel were blinded to treatment assignments."
Incomplete outcome data (attrition bias)<br>All outcomes	Low	1.4% loss to follow up
Selective reporting (reporting bias)	Low	Study protocol was provided and reviewed, no outcomes were added or removed.
Other bias	Low	No other sources of bias were identified.

Table S4e. Rohner *et al* (2010)

Methods	**Trial design**: Double-blind, individually-randomized, placebo-controlled, factorial-design trial of malaria intermittent parasite clearance, antihelminth treatment, and iron fortification<br><br>**Follow-up**: Endpoint assessment<br><br>**Adverse event monitoring**: Not reported
Participants	**Number of participants randomised**: 591<br><br>**Inclusion criteria**: 6- to 14-year-olds; Witten informed consent; Anticipated local residence for the study duration<br><br>**Exclusion criteria**: Major chronic illness; Pregnancy; Known or reported hypersensitivity to study drugs; Anthelmintic treatment within the previous 4 weeks
Interventions	Main trial compared 8-arm factorial design of the following interventions:<br><br>• SP = Sulfadoxine-pyrimethamine 500/25 mg once, administered every three months, two times<br>• I = Biscuits fortified with 20mg iron, administered 4 times/week<br>• AH = Albendazole (400mg) + Praziquantel (40mg/kg) once, administered every three months, two times<br>• P = Placebo (matching parallel drugs and unfortified biscuits)<br><br>All arms containing SP (SP+P+P; SP+I+P; SP+P+AH; SP+I+AH) were pooled and compared to all arms without SP (P+P+P; P+I+P; P+P+AH; P+I+AH) for the meta-analysis.
Outcomes	**Outcomes included in the review**<br><br>• Anaemia (Hb ≤115 g/L for children <12y; ≤120 g/L for children ≥12y by AcT8 Counter)<br>• Parasitaemia (smear)<br><br>**Time (days) from last dose to outcome measurement**: approximately 90
Notes	**Country**: Côte d'Ivoire<br><br>**Setting**: Not reported<br><br>***Pf*PR$_{2\text{-}10}$**: 48-55% (measured *Pf* prevalence in control group at baseline 62%)<br><br>**Coverage**: Not applicable as individually randomized study<br><br>**Estimated proportion of time protected**: 29%<br><br>**Other interventions**: As described anti-helminth treatment and iron fortification, though this was balanced in arms pooled for meta-analysis.<br><br>**Adverse events**: Not reported<br><br>**Drug resistance data**: None<br><br>**Funding**: Medicor Foundation, Swiss Foundation for Research in Nutrition, Hochstrasser Foundation. In-kind contributions: Midor AG, Dafra Pharma, Dr. Lohmann GmbH, Nestle´

Risk of bias		
**Bias**	**Authors' judgement**	**Support for judgement**
Random sequence generation (selection bias)	Low	"children were individually randomized to 1 of the randomization letters in blocks of 8."
Allocation sequence (selection bias)	Low	"codes were held by a member of an independent data safety and monitoring board until data analysis was completed"
Blinding (performance bias and detection bias)<br>All outcomes	Low	Matching placebos used for all study drugs and iron fortified biscuits. Quote: "2 types of biscuits were compared in a triangle test and were indistinguishable by local adults."
Blinding of outcome assessment (detection bias)<br>All outcomes	Low	Communication with authors confirmed outcome assessors were blinded.
Incomplete outcome data (attrition bias)<br>All outcomes	Low	"591 school children were enrolled and 554 completed the study. The main reason for drop-out was out-migration due to a persisting teachers' strike" 6.3% loss to follow-up.
Selective reporting (reporting bias)	Low	Outcomes reported to controlled-trials.com have been analyzed and are published or in-press.
Other bias	Low	Modified intention to treat analysis. Imputed missing values (n=37) with "median values of own group" and excluded participants who did not have baseline measurement.

Table S4f. Clarke *et al* (2012)

Methods	**Trial design**: Double-blind, individually-randomized, placebo-controlled trial of parasite clearance<br><br>**Follow-up**: Follow-up survey 3 months after the intervention<br><br>**Adverse event monitoring**: Active monitoring for three days after each treatment, Passive monitoring for 28 days by school staff and local health centre with treatment costs covered and transport reimbursement
Participants	**Number of participants randomised**: 865<br><br>**Inclusion criteria**: Age 7-14 years; Parental consent available<br><br>**Exclusion criteria**: Chronic conditions which limit regular school attendance; Clinical malaria on the day of scheduled treatment (fever and positive rapid diagnostic test)
Interventions	**Arm 1**: Sulfadoxine-pyrimethamine once + Amodiaquine for three days, administered once (given as annual clearance treatment at end of malaria transmission season)<br><br>**Control**: Placebo
Outcomes	**Outcomes included in the review**<br><br>• Anaemia (Hb<110g/L by HemoCue)<br>• Parasitaemia (smear)<br>• Cognition [tests of sustained attention (code transmission and counting sounds in a cohort of 11-12-year-olds), Raven's matrices]<br><br>**Time (days) from last dose to outcome measurement**: approximately 90
Notes	**Country**: Senegal<br><br>**Setting**: 6 primary schools<br><br>***Pf*PR$_{2\text{-}10}$**: 16%<br><br>**Coverage**: Not applicable as individually randomized study<br><br>**Estimated proportion of time protected**: 63%<br><br>**Other interventions**: National programmes of school-based deworming and universal bed net distribution targeting all age groups (study recorded high net availability and use)<br><br>**Adverse events**: Not available. Personal communication with the investigators "…main side-effects were problems of abdominal discomfort, nausea, and/or vomiting shortly after treatment, fatigue and lethargy in 10-15% of children treated."<br><br>**Drug resistance data**: None<br><br>**Funding**: Wellcome Trust<br>*Only publication is a clinical trial registry and meeting abstract
Risk of bias	

Bias	Authors' judgement	Support for judgement
Random sequence generation (selection bias)	Low	Personal communication with the investigators "…block randomization, using computer generated sequence"
Allocation sequence (selection bias)	Low	Personal communication "placebo-controlled trial, with treatments packaged in numbered envelopes, randomized within blocks, pre-assignment. Researchers and participants were thus blind to study allocation at the time of recruitment and treatment."
Blinding (performance bias and detection bias)<br>All outcomes	Low	Per clinicaltrials.gov, participants and investigators were blinded. Personal communication "participants, investigators, laboratory technicians and analysis (were) blind to study allocation"
Blinding of outcome assessment (detection bias)<br>All outcomes	Low	Per clinicaltrials.gov, outcomes assessors were blinded. Personal communication above
Incomplete outcome data (attrition bias)<br>All outcomes	Unclear	Outcomes data available for 783 of the targeted 860 (91%).
Selective reporting (reporting bias)	Unclear	Unclear as outcomes not published
Other bias	Unclear	Unclear as outcomes not published

Table S4g. Halliday *et al* (2014)

Methods	**Trial design**: Unblinded, Cluster-randomized, Factorial-design trial of a literacy intervention and malaria screening and treatment (results reported separately)<br><br>**Follow-up**: Health outcomes measured by surveys at 12 and 24 months; Education outcome measures at 9 and 24 months<br><br>**Adverse event monitoring**: Study team monitored for 24h hours, passive surveillance system in schools for 28d. Travel costs were reimbursed and treatment charges waived.
Participants	**Number of participants randomised**: 5,233<br><br>**Inclusion criteria**: Enrolled in class 1 or class 5; Provision of informed consent; Child willingness<br><br>**Exclusion criteria**: Allergy or adverse reaction to study medication; Known or suspected to be homozygous for sickle cell trait; Pregnant
Interventions	**Arm 1**: Screening with RDT, if positive treated with weight-based dosing Artemisinin-lumefantrine twice daily for three days (doses 1, 3, 5 observed), once per school term, 5 times<br><br>**Arm 2**: Literacy intervention<br><br>**Arm 3**: Both interventions<br><br>**Control**: No intervention
Outcomes	**Outcomes included in the review**<br><br>• Cognition (code transmission test for older, pencil tap test for younger)<br>• Parasitaemia (smear)<br>• Educational achievement (tests of literacy and numeracy)<br><br>**Time (days) from last dose to outcome measurement**: approximately 150 days
Notes	**Country**: Kenya<br><br>**Setting**: 101 government primary schools<br><br>***Pf*PR$_{2\text{-}10}$**: 3-13% (measured *Pf* prevalence in intervention group at baseline 13%, not measured in control group)<br><br>**Coverage**: 66.8% (received screening at all 5 rounds)<br><br>**Estimated proportion of time protected**: 2%<br><br>**Other interventions**: albendazole was delivered through households as part of the national lymphatic filariasis campaign in 2011, although coverage was not extensive and praziquantel was delivered to schools in the area in June 2011.<br><br>**Adverse events**: 4.5% reported one or more adverse effects within 2 days of treatment. 11 children died: five in intervention and six in the control. None of these deaths occurred within 30 days of the screening and treatment and were not attributed to the intervention.<br><br>**Drug resistance data**: None

	**Funding**: International Initiative for Impact Evaluation, Partnership for Child Development, World Bank, Wellcome Trust	
Risk of bias		
**Bias**	**Authors' judgement**	**Support for judgement**
Random sequence generation (selection bias)	Low	"IST intervention was randomly allocated at the level of the school, with the 101 schools re-stratified by (i) literacy intervention group assignment and (ii) quintiles of average school exam scores, producing ten strata overall."
Allocation sequence (selection bias)	Low	Not applicable based on study design
Blinding (performance bias and detection bias)<br>All outcomes	High	Not blinded based on design of the intervention
Blinding of outcome assessment (detection bias)<br>All outcomes	Low	"blood slides were read independently by two microscopists who were blinded to group allocation."
Incomplete outcome data (attrition bias)<br>All outcomes	Low	"4,201 (80.3%) were included in the 24-month health survey ... children lost to follow-up across both study arms were largely similar to children followed up."
Selective reporting (reporting bias)	Low	Reported primary and secondary outcomes of interest to this review are the same as reported on clinicaltrials.gov
Other bias	Low	No other sources of bias were identified

Table S4h. Nankabirwa *et al* (2014)

Methods	**Trial design**: Double-blind, individually-randomised, placebo-controlled trial of intermittent preventive treatment<br><br>**Follow-up**: Blood smears monthly. History, physical exam, and Hb measured at the beginning of each school term. Absent children were followed up at home. Active case detection by teacher and referral to the study clinic<br><br>**Adverse event monitoring**: All doses were directly observed, adverse events were assessed and graded
Participants	**Number of participants randomised**: 740<br><br>**Inclusion criteria**: Enrolled in study school; Informed consent<br><br>**Exclusion criteria**: Known allergy or adverse reaction to artemisinin-based regimens; Menarche; Fever (>=37.5°C) or history of fever in the last 24h; evidence of severe malaria or danger signs; currently receiving anti-malaria treatment
Interventions	**Arm 1**: Dihydroartemisinin-piperaquine weight-based daily dosing for three days, administered every school term, four times<br><br>**Arm 2**: Dihydroartemisinin-piperaquine weight-based daily dosing for three days, administered monthly, twelve times<br><br>**Control**: Placebo (administered to the control arm and Arm 1 monthly to simulate Arm 2)
Outcomes	**Outcomes included in the review**<br><br>• Clinical malaria (fever or history of fever + positive smear;)<br>• Parasitaemia (smear)<br>• Anaemia (Hb<11.5 g/dL for ages 6 to 11 years; Hb <12.0 g/dL for ages 12 to 14 years; HemoCue)<br>• Cognition (sustained attention by code transmission test; abstract reasoning by Ravens matrices)<br><br>**Time (days) from last dose to outcome measurement**: approximately 30
Notes	**Country**: Uganda<br><br>**Setting**: 1 primary school<br><br>***Pf*PR$_{2\text{-}10}$**: 42% (measured *Pf* prevalence in control group at baseline 32%)<br><br>**Coverage**: Not applicable as individually randomized study<br><br>**Estimated proportion of time protected**: 30% for DP termly, 90% for DP monthly<br><br>**Other interventions**: Mass net distribution the month prior to study start, all enrolled children received a LLIN and a single dose of albendazole<br><br>**Adverse events**: 1 death, due to acute lymphoblastic leukaemia, in IPTst. 14 SAEs (6 in Arm 2; 5 in Arm 1; 3 in the Control arm) none were judged unlikely to be associated with treatment; mild events were more frequent in the placebo group than the intervention arms.

	**Drug resistance data**: Mutations at *pfmdr1* N86Y and *pfcrt* K76T were more prevalent among samples from participants treated with DP in the last 30 days than samples from those not treated with DP in the last 60 days.<br><br>**Funding**: Malaria Capacity Development Consortium, Bill & Melinda Gates Foundation, Fogarty International Centre of the National Institutes of Health, Wellcome Trust, Holley-Cotec provided the dihydroartemisinin-piperaquine	
Risk of bias		
**Bias**	**Authors' judgement**	**Support for judgement**
Random sequence generation (selection bias)	Low	"A randomization list was computer generated using fixed blocks of 12 by an individual not involved in patient care."
Allocation sequence (selection bias)	Low	"Participants were randomized using previously prepared, consecutively numbered, opaque, sealed envelopes and assigned study numbers sequentially. A nurse not involved in care allocated the study group after opening the correspondingly numbered envelope."
Blinding (performance bias and detection bias)<br>All outcomes	High	"All other study personnel were blinded to study group assignments, and children were not informed of their regimen, but the colour and taste of study medication and placebo were dissimilar."
Blinding of outcome assessment (detection bias)<br>All outcomes	Low	"Our outcome measures included clinical and laboratory evaluations by study personnel blinded to the participants' study arms."
Incomplete outcome data (attrition bias)<br>All outcomes	Low	Of 740, only 27 participants were lost (3.6%). Distribution was relatively equal among groups.
Selective reporting (reporting bias)	High	Outcomes reported to clinicaltrials.gov were reviewed. Education outcomes have thus far not been published.
Other bias	Low	No other sources of bias were identified.

Table S4i. Opoku *et al* (2016)

Methods	**Trial design**: open-label, individually-randomized trial of malaria intermittent preventive treatment combined with antihelminth treatment<br><br>**Follow-up**: Evaluation 12 months after enrolment<br><br>**Adverse event monitoring**: Active and passive follow-up to 28 days after treatment	
Participants	**Number of participants randomised**: 360<br><br>**Inclusion criteria**: Enrolled in Class 3; Parental consent provided<br><br>**Exclusion criteria**: Known history of allergy or adverse reaction to any study medications; Sick	
Interventions	**Arm 1**: Artemether-lumefantrine (20mg/120mg) twice daily for three days, administered every 3 months, three times + Albendazole (400mg) administered every 6 months, two times<br><br>**Arm 2**: Artemether-lumefantrine (20mg/120mg) twice daily for three days, administered every 3 months, three times + Albendazole (400mg), administered every 6 month, two times + Praziquantel (40mg/kg), once<br><br>**Control**: Albendazole (400mg), administered every 6 month, two times + Praziquantel (40mg/kg), once	
Outcomes	**Outcomes included in the review**<br><br>• Parasitaemia (smear)<br>• Anaemia (Hb<11.0g/dL; HemoCue)<br>• Cognition (code transmission test; recall testing)<br><br>**Time (days) from last dose to outcome measurement**: approximately 180	
Notes	**Country**: Ghana<br><br>**Setting**: 6 primary schools<br><br>***Pf*PR$_{2\text{-}10}$**: 65%<br><br>**Coverage**: Not applicable as individually randomized study<br><br>**Estimated proportion of time protected**: 12%<br><br>**Other interventions**: Antihelminth treatment to all<br><br>**Adverse events**: Not reported<br><br>**Drug resistance data**: None<br><br>**Funding**: Danish International Development Agency	
Risk of bias		
**Bias**	**Authors' judgement**	**Support for judgement**

Random sequence generation (selection bias)	Low	Purposive sampling of 6 schools near health center, 6 were randomized to 3 arms
Allocation sequence (selection bias)	Low	One randomization exercise
Blinding (performance bias and detection bias)<br>All outcomes	High	Not blinded
Blinding of outcome assessment (detection bias)<br>All outcomes	Unclear	No description of blinding of outcome assessment
Incomplete outcome data (attrition bias)<br>All outcomes	Low	97% follow-up
Selective reporting (reporting bias)	Low	Reported primary and secondary outcomes of interest to this review are the same as reported on clinicaltrials.gov
Other bias	Low	No other sources of bias were identified.

Table S4j. Clarke *et al* (2017)

Methods	**Trial design**: Single-blind, cluster-randomized trial of malaria education, LLIN, and parasite clearance<br><br>**Follow-up**: Cross-sectional surveys in a subset of schools for health outcomes (preintervention, between net distribution and parasite clearance, and two after parasite clearance). Cognition outcomes measured in a longitudinal cohort of 9- to 12-year olds<br><br>**Adverse event monitoring**: AEs were monitored by teachers for 7 days after treatment and referred to the health centre	
Participants	**Number of participants in follow-up surveys**: 1861<br><br>**Inclusion criteria**: Enrolled in study school<br><br>**Exclusion criteria**: History of adverse reaction to sulfa-based drugs; Known or suspected pregnancy (not tested); Concurrent treatment for clinical malaria	
Interventions	**Intervention**: Malaria prevention education and two LLINs to school children at the beginning of the transmission season, Sulfadoxine-pyrimethamine one age-based dose + Artesunate age-based doing daily for three days, administered once as annual clearance treatment at the end of the transmission season<br><br>**Control**: LLINs through national net distribution programme	
Outcomes	**Outcomes included in the review**<br><br>• Anaemia (WHO age-gender cut-offs; HemoCue)<br>• Parasitaemia (smear)<br>• Cognition (sustained attention – code transmission, visual search, numeracy, vocabulary, and writing)<br><br>**Time (days) from last dose to outcome measurement:** approximately 60	
Notes	**Country**: Mali<br><br>**Setting**: 80 primary schools<br><br>***Pf*PR$_{2\text{-}10}$**: 50-67% (measured *Pf* prevalence in control group at baseline 78%)<br><br>**Coverage**: 94.6% (3 days of treatment, 1 treatment round)<br><br>**Estimated proportion of time protected**: 58%<br><br>**Other interventions**: LLINs as described above, national deworming programme<br><br>**Adverse events**: Few AEs: mild, self-limited stomach ache and vomiting after treatment. No SAEs were reported.<br><br>**Drug resistance data**: None<br><br>**Funding**: Save the Children, Wellcome Trust	
Risk of bias		
**Bias**	**Authors' judgement**	**Support for judgement**

Random sequence generation (selection bias)	Low	Random selection within strata, then random assignment to intervention or control
Allocation sequence (selection bias)	Low	Not applicable based on study design
Blinding (performance bias and detection bias)<br>All outcomes	High	Not blinded based on open-label design of the intervention
Blinding of outcome assessment (detection bias)<br>All outcomes	Low	"Staff responsible for measuring study outcomes were unaware of group allocation. Data analysis was undertaken by a statistician with no prior involvement in the trial."
Incomplete outcome data (attrition bias)<br>All outcomes	Low	Biomedical outcomes assessed in cross-sectional survey of a random-selected sub-sample of children (all classes) drawn on the day of the survey; 50 per school, 950 per study arm
Selective reporting (reporting bias)	Low	Outcomes as planned on clinicaltrails.com, though absenteeism removed as secondary outcome
Other bias	Low	No other sources of bias were detected.

Table S4k. Matangila *et al* (2017)

Methods	**Trial design**: Open-label, individually-randomized trial of intermittent preventive treatment<br><br>**Follow-up**: Prevalence surveys at 4, 7, and 12 months after enrolment. Clinical malaria incidence only followed for 4 months after enrolment<br><br>**Adverse event monitoring**: Case report forms filled details of active vs passive surveillance not provided
Participants	**Number of participants randomised**: 616<br><br>**Inclusion criteria**: Enrolled in primary school years 1-5; Parental consent and if applicable assent<br><br>**Exclusion criteria**: Participation in another drug study in the last 30d; Known or suspected hypersensitivity or serious AE to study drugs; Malaria symptoms at baseline; Fever at baseline; Decompensated anaemia; Other illness limiting study participation including G6PD deficiency and sickle cell; Weight <14kg; Other chronic infectious diseases, e.g. HIV and TB
Interventions	**Arm 1**: Sulfadoxine-pyrimethamine one weight-based dose, administered every 4 months, three times<br><br>**Arm 2**: Sulfadoxine-pyrimethamine one weight-based dose + Piperaquine weight-based dosing daily for two days, administered every 4 months, three times<br><br>**Control**: No treatment
Outcomes	**Outcomes included in the review**<br><br>• Anaemia (Hb<11, <11.5 and <12 g/dL, respectively, for ages <5 years, 5– to 11.9-years and 12- to 14.9-years; Hemo Control)<br>• Parasitaemia (smear)<br>• Clinical malaria incidence (passive surveillance)<br><br>**Time (days) from last dose to outcome measurement**: approximately 150
Notes	**Country**: Democratic Republic of Congo<br><br>**Setting**: 2 primary schools<br><br>***Pf*****PR$_{2-10}$**: 16% (measured *Pf* prevalence in control group at baseline 19%)<br><br>**Coverage**: Not applicable as individually randomized study<br><br>**Estimated proportion of time protected**: 29%<br><br>**Other interventions**: All participants received praziquantel and albendazole at enrolment.<br><br>**Adverse events**: No deaths or SAEs. Frequency of any AE was higher SP/PQ compared with controls (P = 0.0069). Dizziness was associated with SP/PQ (P= 0.0025). When excluding dizziness, frequency of any AEs did not differ between treatment arms.

	**Drug resistance data**: Published protocol states plan to measure prevalence of DHFR and DHPS mutations at baseline and after 12 months. However, these analyses were not undertaken (personal communication).<br><br>**Funding**: VLIR UOS Project, FWO. Drugs provided free of charge by Sigma Tau, IDA Foundation	
Risk of bias		
**Bias**	**Authors' judgement**	**Support for judgement**
Random sequence generation (selection bias)	Low	"randomly assigned ... according to a predetermined randomization list of blocks of eight generated by a statistician."
Allocation sequence (selection bias)	Low	"Sealed envelopes labelled with the school unique code and containing the list with the treatment allocated to the participants will be provided according to the above mentioned list and opened at the moment the children are recruited."
Blinding (performance bias and detection bias)<br>All outcomes	High	Open-label
Blinding of outcome assessment (detection bias)<br>All outcomes	Low	Personal communication with the authors. Outcome assessors were blinded.
Incomplete outcome data (attrition bias)<br>All outcomes	High	Present only per protocol and modified intention to treat analyses due to incomplete follow up
Selective reporting (reporting bias)	Low	Primary outcome is reported as described in Trials manuscript.
Other bias	Low	No other sources of bias were identified.

Table S4l. Rehman et al (2019) and Staedke et al (2018)

Methods	**Trial design**: Open-label, cluster-randomized trial of intermittent preventive treatment<br><br>**Follow-up**: Cross-sectional school-based survey at the end of the intervention period. Random selection of sub-sample of students in each intervention and control school. Community-level parasitaemia prevalence and EIR also conducted to measure indirect outcomes<br><br>**Adverse event monitoring**: All intervention participants were monitored for SAEs. Subset of participants selected by convenience sampling for cardiac monitoring
Participants	**Number of participants in follow-up survey**: 1092<br><br>**Inclusion criteria**: Enrolled in intervention school; age ≥ 5-years; Parental consent; Student assent (if ≥ 8-years-old)<br><br>**Exclusion criteria**: Known allergy to study drug; Menarche; Family history of long-QT; Taking other medications known to prolong QT interval; Weight <11kg
Interventions	**Arm 1**: Dihydroartemisinin-piperaquine weight-based dosing daily for three days, administered monthly, six times<br><br>**Control**: No treatment
Outcomes	**Outcomes included in the review**<br><br>• Parasitaemia (smear)<br>• Anaemia (HemoCue)<br>• Clinical malaria (prevalence – fever (>=38°C) or history of fever in last 48h AND positive RDT) **was not a prespecified outcome and was measured only as active case detection in the final survey and, thus, not included in the meta-analysis.<br><br>**Time (days) from last dose to outcome measurement**: 1 to 113 days (mean 15.3 days; SD 16 days)
Notes	**Country**: Uganda<br><br>**Setting**: 84 primary schools<br><br>***Pf*PR$_{2\text{-}10}$**: 7-16% (measured *Pf* prevalence in <5yo in control group at baseline 26%) Coverage (participants in the intervention arm in the school surveys received treatment in all six rounds): 7.1%<br><br>**Estimated proportion of time protected**: 49%<br><br>**Other interventions**: None<br><br>**Adverse events**: Unpublished to date, but results reported in the WHO Evidence Review Group on the cardiotoxicity of antimalarials (https://www.who.int/malaria/mpac/mpac-mar2017-erg-cardiotoxicity-report-session2.pdf)<br><br>**Drug resistance data**: None.<br><br>**Funding**: UK Medical Research Council, UK Department for International Development, and Wellcome Trust

Risk of bias		
**Bias**	**Authors' judgement**	**Support for judgement**
Random sequence generation (selection bias)	Low	"used restricted randomisation to ensure balance across clusters for geographical location by subcounty and school type (public or private)."
Allocation sequence (selection bias)	Low	Not applicable based on study design
Blinding (performance bias and detection bias)<br>All outcomes	High	Not blinded based on design of the intervention
Blinding of outcome assessment (detection bias)<br>All outcomes	Low	"read by experienced laboratory technologists who were unaware of study group assignments."
Incomplete outcome data (attrition bias)<br>All outcomes	Low	School-based outcomes were cross-sectional surveys after the intervention. Sample sizes were generated from baseline data collection.
Selective reporting (reporting bias)	Low	Reported primary and secondary outcomes of interest to this review are the same as reported on clinicaltrials.gov
Other bias	Low	No other sources of bias were identified.

Table S4m. Thera et al (2018)

Methods	**Trial design**: Open-label, individually-randomised trial of intermittent preventive treatment<br><br>**Follow-up**: Monthly active follow-up with physical exam, Hb and smear. Passive surveillance by study staff in the school for the duration of the study<br><br>**Adverse event monitoring**: Active monitoring on days 0, 1, and 3	
Participants	**Number of participants randomised**: 200<br><br>**Inclusion criteria**: Age 6- to 15-years; Enrolled in the study school; Agee to comply with procedure; Parental consent<br><br>**Exclusion criteria**: Known allergic reaction to study drugs; Hb<10g/dL, low blood sugar (< 70mg/dL); Positive malaria smear at screening; Presence of acute illness (danger signs): Chronic illness; Taking anti-malarial drugs in the last 15 days	
Interventions	**Arm 1**: Artesunate + Amodiaquine daily for three days, monthly, four times<br><br>**Control**: No treatment	
Outcomes	**Outcomes included in the review**<br><br>• Clinical malaria (symptoms + positive RDT)<br>• Parasitaemia (smear)<br>• Anaemia (Hb<10g/dL; HemoCue)<br><br>**Time (days) from last dose to outcome measurement**: approximately 90d	
Notes	**Country**: Mali<br><br>**Setting**: 1 primary school<br><br>***Pf*PR$_{2-10}$**: 40%<br><br>**Coverage**: Not applicable as individually randomized study<br><br>**Estimated proportion of time protected**: 32%<br><br>**Other interventions**: None<br><br>**Adverse events**: Most common solicited AEs were abdominal pain (64% of children receiving ASAQ), headaches (44%), dizziness (22%), nausea (7%), and vomiting (6%). No SAEs are reported.<br><br>**Drug resistance data**: None<br><br>**Funding**: Centre National de la Recherche Scientifique et Technologique. Study drugs were donated by Guilin Pharmaceutical Co., Ltd.	
Risk of bias		
**Bias**	**Authors' judgement**	**Support for judgement**
Random sequence generation (selection bias)	Low	"Randomization to study arms was done using a computer-generated randomization list. The randomization list contained sequential codes that linked a study number to an

		arm assignment. Study numbers were assigned to children in the order in which they were enrolled in the trial."
Allocation sequence (selection bias)	High	"There were no masking procedures of study products assignments."
Blinding (performance bias and detection bias)<br>All outcomes	High	Not blinded based on study design
Blinding of outcome assessment (detection bias)<br>All outcomes	High	Outcomes assessors for point-of-care assessments (anemia, clinical symptoms, and malaria rapid diagnostic test) were aware of the participant study arm.
Incomplete outcome data (attrition bias)<br>All outcomes	Low	"In the ASAQ arm, two participants were excluded after randomization; one because of an adverse event due to ASAQ and reported by his father and the second for non-compliance with study visits schedule. In the control arm, one participant was excluded for non-compliance with the study visits schedule." All participants were included in the analysis
Selective reporting (reporting bias)	Low	All measured outcomes were reported based on the Pan African Clinical Trials Registry. Acceptability and school success outcomes were planned but not measured.
Other bias	Low	No other sources of bias were identified.

Table S5. Between-study variance explained by study-level characteristics in meta-regression

Outcome	Study level characteristics				
	**$PfPR_{2\text{-}10}$***	**Drug type (ACT vs non-ACT)**	**Region (East and Central vs. West Africa)**	**Protected time**	**Study design (individually vs. cluster randomized)**
Plasmodium falciparum infection	-20.0%<br>p = 0.919	-5.4%<br>p = 0.574	-0.9%<br>p = 0.365	3.4%<br>p = 0.254	-3.1%<br>p = 0.441
Anaemia	-16.7%<br>p = 0.863	-11.1%<br>p = 0.958	-9.8%<br>p = 0.623	7.1%<br>p = 0.419	-5.6%<br>p = 0.533
Clinical malaria during follow-up	0.0%<br>p = 0.677	-70.7%<br>p = 0.908	-75.3%<br>p = 0.659	48.2%<br>p = 0.351	_†

* *Plasmodium falciparum* prevalence among children 2-10 years
† There were no cluster randomized trials reporting clinical malaria as an outcome

Table S6. Intervention effects stratified by age group

Outcome	Effect in age group (95%CI)		p-value for interaction
	**RR in under 10-year-olds**	**RR in 10-year-olds and older**	
Plasmodium falciparum infection†	0.47 (0.40 – 0.54)	0.45 (0.38 – 0.52)	0.606
Anaemia†	0.79 (0.71 – 0.88)	0.91 (0.81 – 1.00)	0.015
Clinical malaria during follow-up‡	0.60 (0.45 – 0.75)	0.35 (0.22 – 0.49)	0.067
	**Difference in under 10-year-olds**	**Difference in 10-year-olds and older**	
Code transmission test scores§	-0.33 (-0.77 – 0.11)	0.36 (0.01 – 0.71)	0.004

RR = Risk ratios that were obtained by marginal standardization; p-values from corresponding logistic regression and adjusted for sex or age, as relevant, treatment group and transmission intensity

† Eleven studies contributing 15,658 observations, with 6,908 less than 10 years old and 8,635 aged 10 or older and 115 missing age

‡ Four studies contributing 1,815 observations, with 889 less than 10 years old and 747 aged 10 or older and 179 missing age

§ Five studies contributing 6,066 observations, with 2,101 less than 10 years old and 3,962 aged 10 or older and 3 missing age

Table S7. Effect of treatment on prevalence *Plasmodium falciparum* infection stratified by transmission setting

**Transmission setting (*Pf*PR$_{2-10}$)**	**Adjusted risk ratio for *P. falciparum* infection**	**95% CI**	**p-value**
Low (<10%)	0.51	0.33 – 0.69	<0.001
Low-moderate (10% to <30%)	0.36	0.27 – 0.45	<0.001
Moderate-high (30% to <50%)	0.52	0.43 – 0.60	<0.001
High (≥50%)	0.44	0.25 – 0.63	<0.001

***Pf*PR$_{2-10}$ is estimated from the Malaria Atlas Project, see Methods for details.**
There is evidence of interaction between treatment and transmission intensity on effect on *P. falciparum* infection (LRT p-value = 0.0001); however, there is no consistent pattern to this interaction suggesting perhaps the additional role of the different drug types in the effect estimates. The data are too sparse to investigate the joint effects of drugs and transmission intensity on effect (Table S8). There is no evidence of interaction effect on anaemia or clinical malaria. There was insufficient variation in transmission setting among the studies that measured cognitive function using code transmission testing to allow evaluation of the effect of treatment on that outcome by transmission setting.

Table S8. Distribution of overall sample by drug type and malaria transmission setting

Drug type	Transmission setting (*Pf*PR_{2-10}))				Total
	Low (<10%)	Low to moderate (10% to <30%)	Moderate to high (30% to <50%)	High (≥50%)	
Control group	1,422	1,959	2,886	954	7,221
Artemether-lumefantrine	1,580	559	0	0	2,139
Artesunate plus SP	96	0	100	797	993
ACT containing aminoquinoline	190	444	764	0	1,398
SP plus aminoquinoline	0	984	2,320	0	3,304
SP	0	139	413	51	603
**Total**	**3,288**	**4,085**	**6,483**	**1,802**	**15,658**

Note: *Pf*PR_{2-10} is estimated from the Malaria Atlas Project, see Methods for details.

Table S9. Sensitivity analysis: Effect of antimalarial intermittent preventative treatment on primary and secondary outcomes excluding Halliday *et al.* 2014 and Rehman *et al*. 2019 (school-based results from the main study published as Staedke *et al*. 2018)

**Outcome**	**Control, n (%)**	**Intervention, n (%)**	**Crude risk ratio* (95%CI)**	**p-value**	**Adjusted† risk ratio (95% CI)**	**p-value**
Plasmodium falciparum infection	2,117 (45.2%)	567 (9.8%)	0.45 (0.37 – 0.54)	<0.001	0.42 (0.33 – 0.50)	<0.001
Anaemia	1,069 (22.9%)	924 (15.9%)	0.80 (0.71 –0.88)	0.001	0.79 (0.70 – 0.87)	0.001
Clinical malaria during follow-up‡	144 (24.8%)	134 (12.7%)	0.56 (0.45 – 0.67)	< 0.001	0.50 (0.39 – 0.60)	<0.001
	**Control, mean (SE)**	**Intervention, mean (SE)**	**Crude difference (95%CI)**	**p-value**	**Adjusted† difference (95% CI)**	**p-value**
Code transmission test scores§	11.86 (0.17)	12.37 (0.15)	0.31 (-0.09 – 0.71)	0.127	0.16 (-0.24 – 0.57)	0.421

There are 4,680 observations in the control arms and 5,760 in the intervention arms for analyses of *Pf* infection and anaemia when Halliday 2014 and Rehman 2019 are excluded
*Risk ratios are obtained by marginal standardization; p-values from corresponding logistic regression
†Adjusted for age, sex and transmission intensity
‡Four studies contributing 637 individuals in the control arm and 1,178 in the intervention arm
§Four studies (excluding Halliday 2014 data) contributing 979 individuals in the control arm and 1,255 in the intervention arm

Figure S1. Impact of proportion of follow-up time protected by treatment on *Plasmodium falciparum* infection by transmission setting

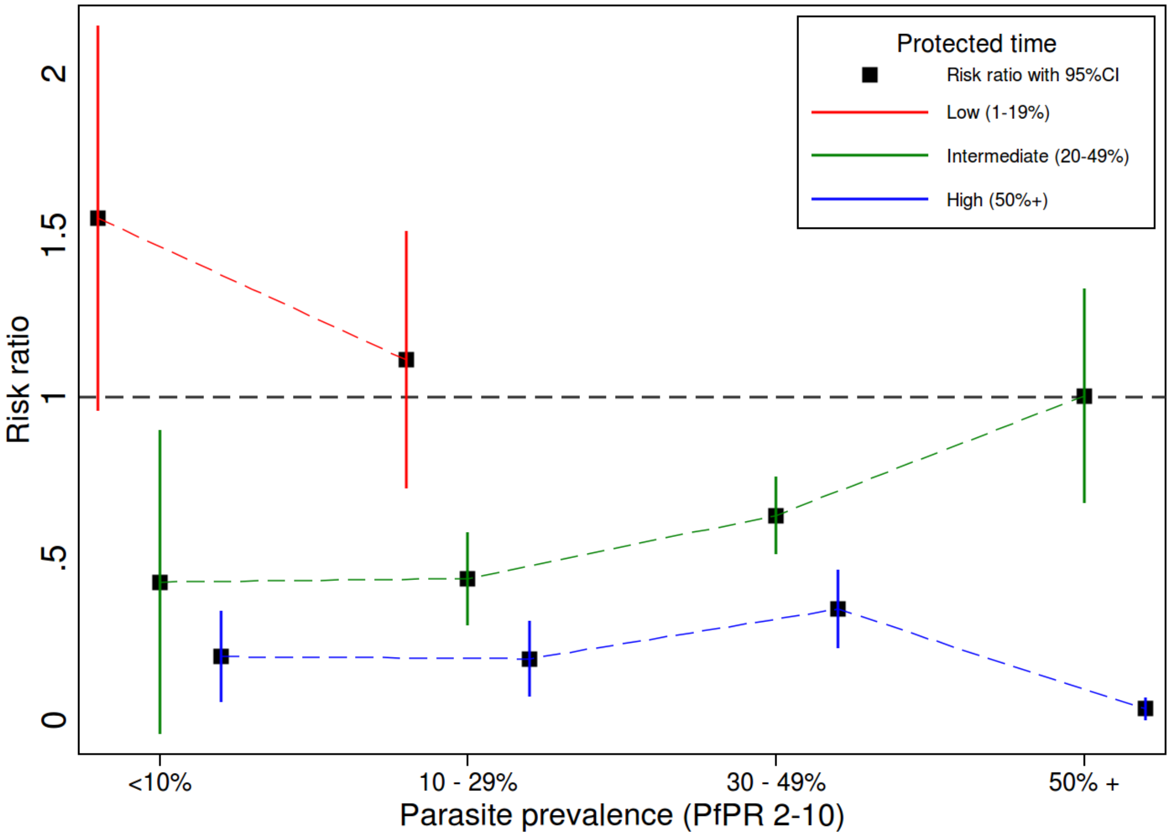

Interpretation: The risk ratio drug interventions with an intermediate protected time increases as transmission increases and approaches 1.0 in high transmission settings. **Notes:** (1) Black boxes along the X-axis have been spaced so as not to overlap; (2) Dashed lines show the trend in each level of percent protected time.

Figure S2. Forest plot of treatment on clinical malaria by study: Study-level meta-analysis fixed and random effects analyses.

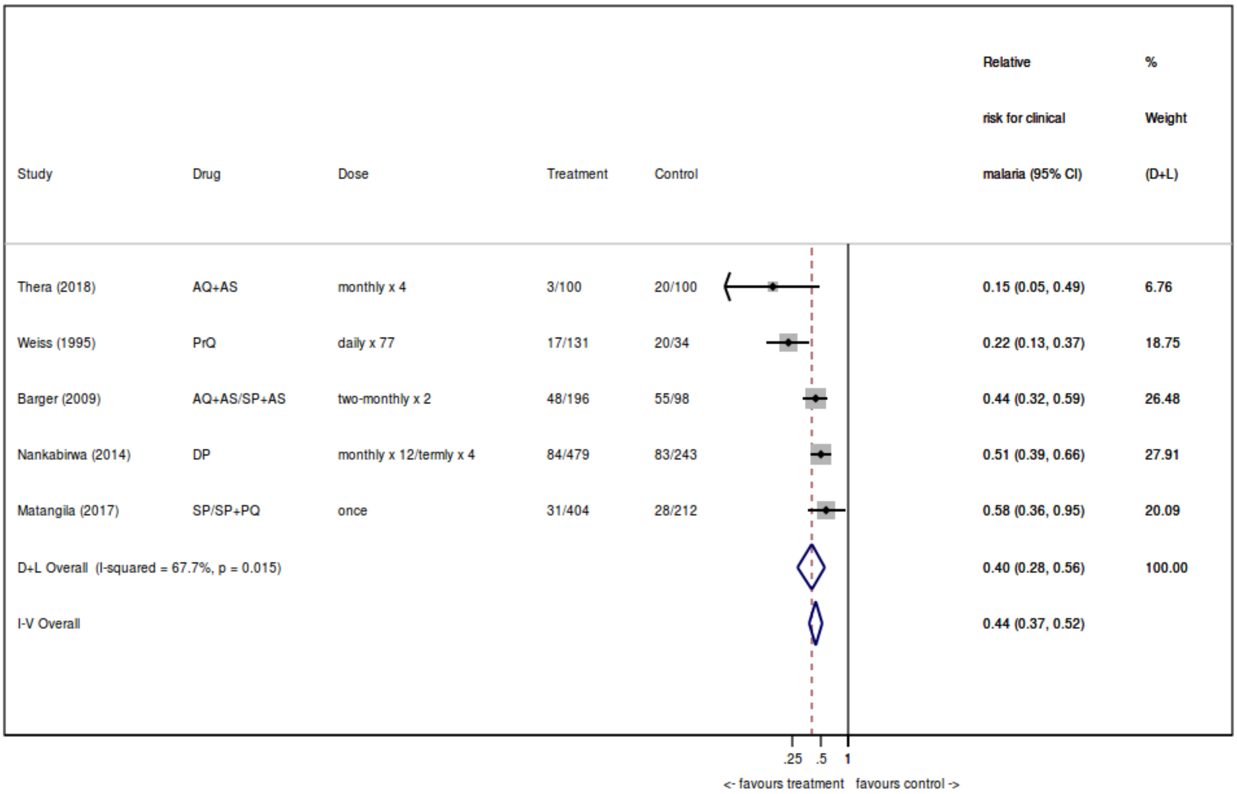

Note: D+L is the pooled random effects estimate and I-V the pooled fixed effects estimate.

Figure S3. Forest plot of treatment on clinical malaria by study drug type: Individual participant meta-analysis (n=; 4 studies, 1,815 individuals)

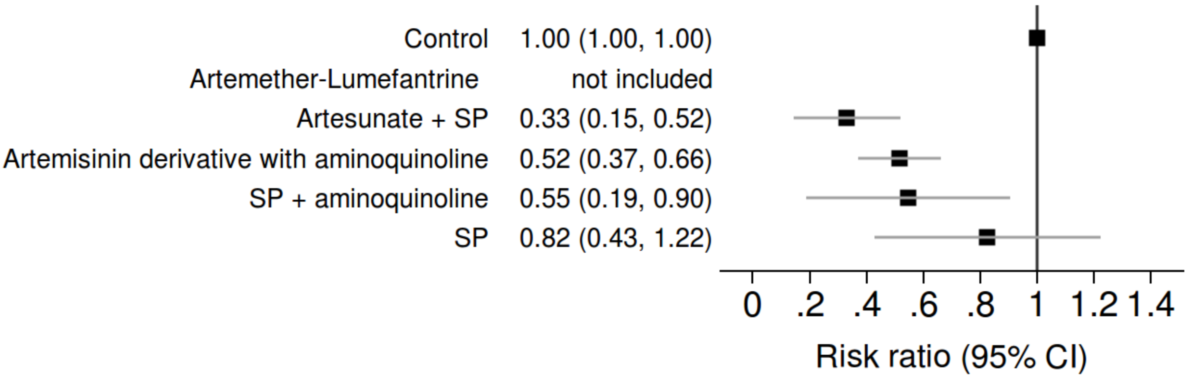

Note: Includes data from Barger *et al*., Nankabirwa *et al*. 2014, Matangila *et al*., Thera *et al*.

Figure S4. Forest plot of code transmission test by study drug type: Individual participant meta-analysis (n=; 5 studies, 6,066 individuals)

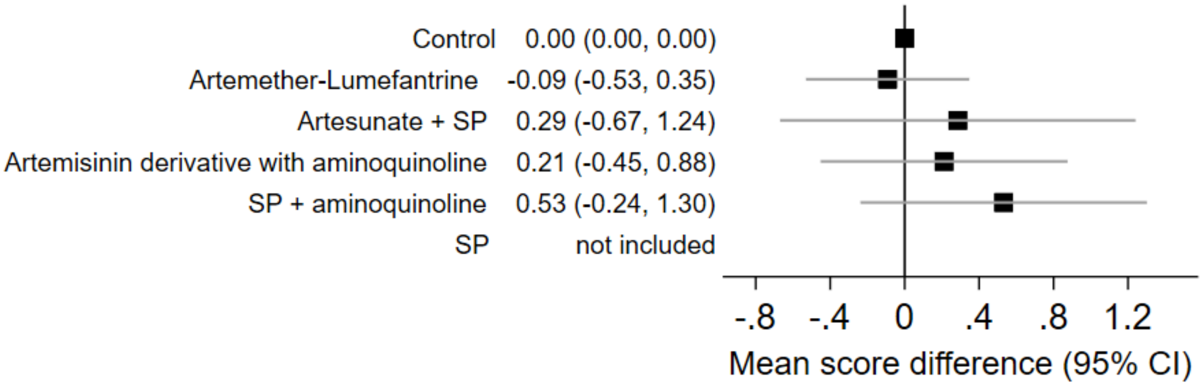

Note: Includes data from Clarke *et al.* 2008, Clarke *et al.* 2017, Clark *et al.* 2012, Halliday *et al*., Nankabirwa *et al*. 2014

Figure S5. Risk of bias within studies

	Random sequence generation (selection bias)	Allocation concealment (selection bias)	Blinding of participants and personnel (performance bias)	Blinding of outcome assessment (detection bias)	Incomplete outcome data (attrition bias)	Selective reporting (reporting bias)	Other bias
Barger, 2009	+	+	−	−	+	+	+
Clarke, 2008	+	+	−	+	+	+	+
Clarke, 2012	+	+	+	+	?	?	?
Clarke, 2017	+	+	−	+	+	+	+
Halliday, 2014	+	+	−	+	+	+	+
Matangila, 2017	+	+	−	+	−	+	+
Nankabirwa, 2010	+	+	−	+	+	+	+
Nankabirwa, 2014	+	+	−	+	+	−	+
Opoku, 2016	+	+	−	?	+	+	+
Rohner, 2010	+	+	+	+	+	+	+
Staedke, 2018	+	+	−	+	+	+	+
Thera, 2018	+	−	−	−	+	+	+
Weiss, 1995	?	?	?	+	+	?	+

Figure S6. Risk of bias across studies

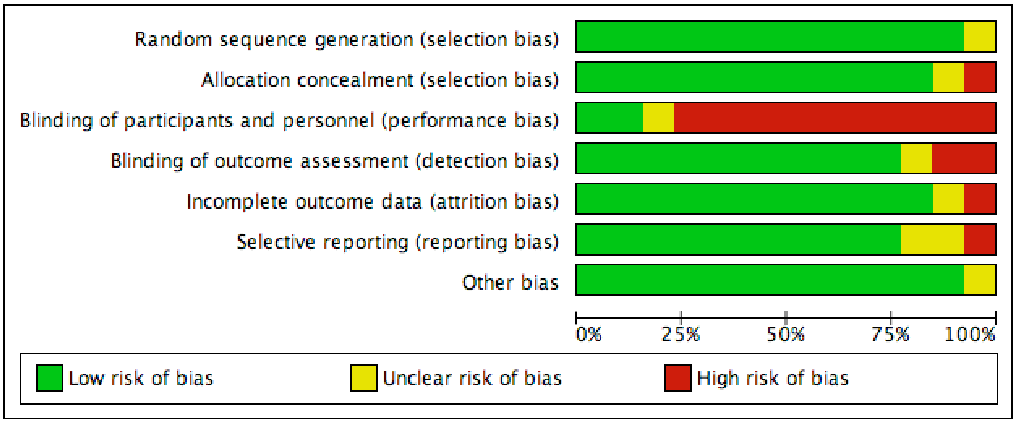

Funnel Plot Interpretation

Funnel plots in Figure S7, Figure S8, and Figure S9 illustrate that there is no pattern of absence of studies that contain either favourable or unfavourable results and no pattern of association between study size and effect size in the funnel plots, suggesting no indication of publication bias. The spread of magnitudes of effect across studies, particularly for parasitaemia, was consistent with high heterogeneity in between-study estimates of effect.

Figure S7. Funnel plot for random effects analysis of *Plasmodium falciparum* infection.

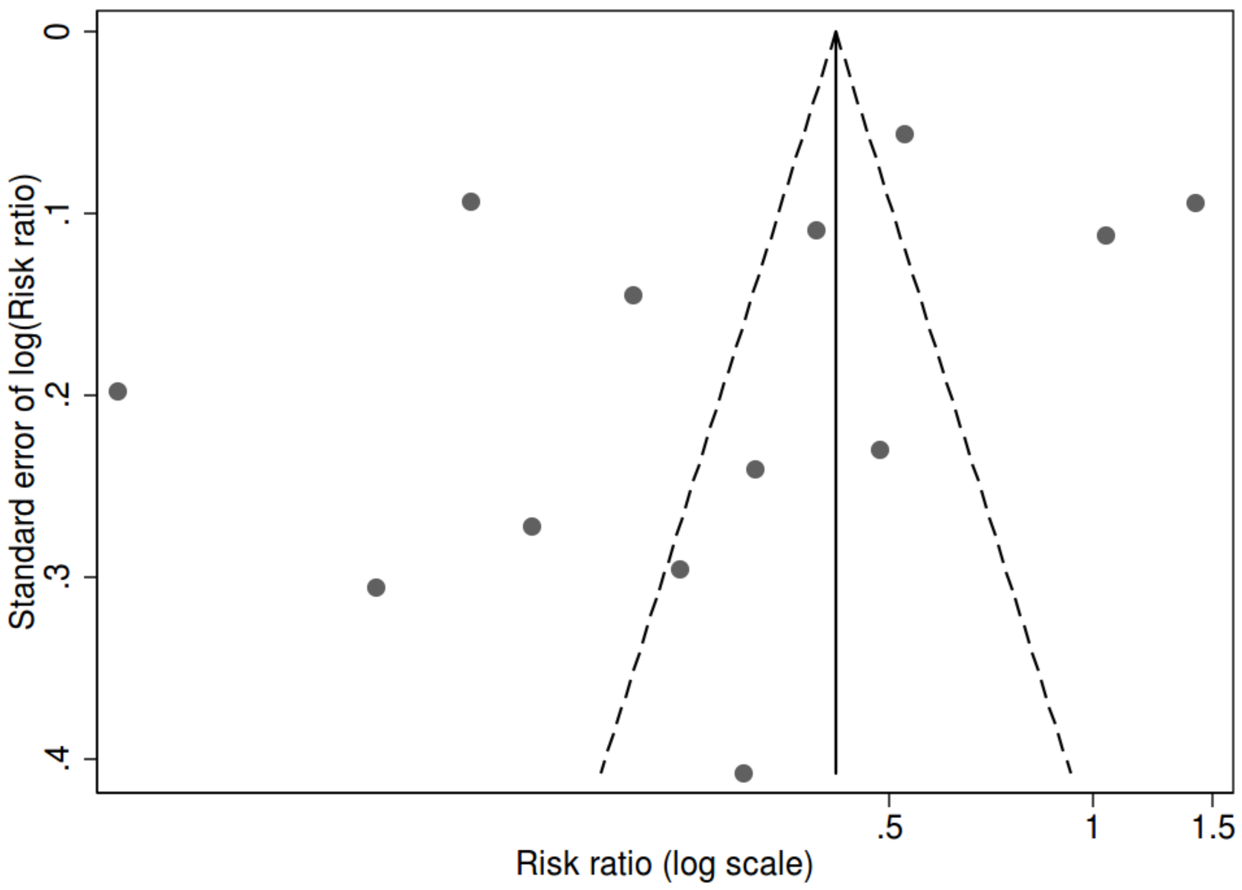

Note: Dotted lines represent pseudo 95% confidence intervals.

Figure S8. Funnel plot of random effects analysis of anaemia.

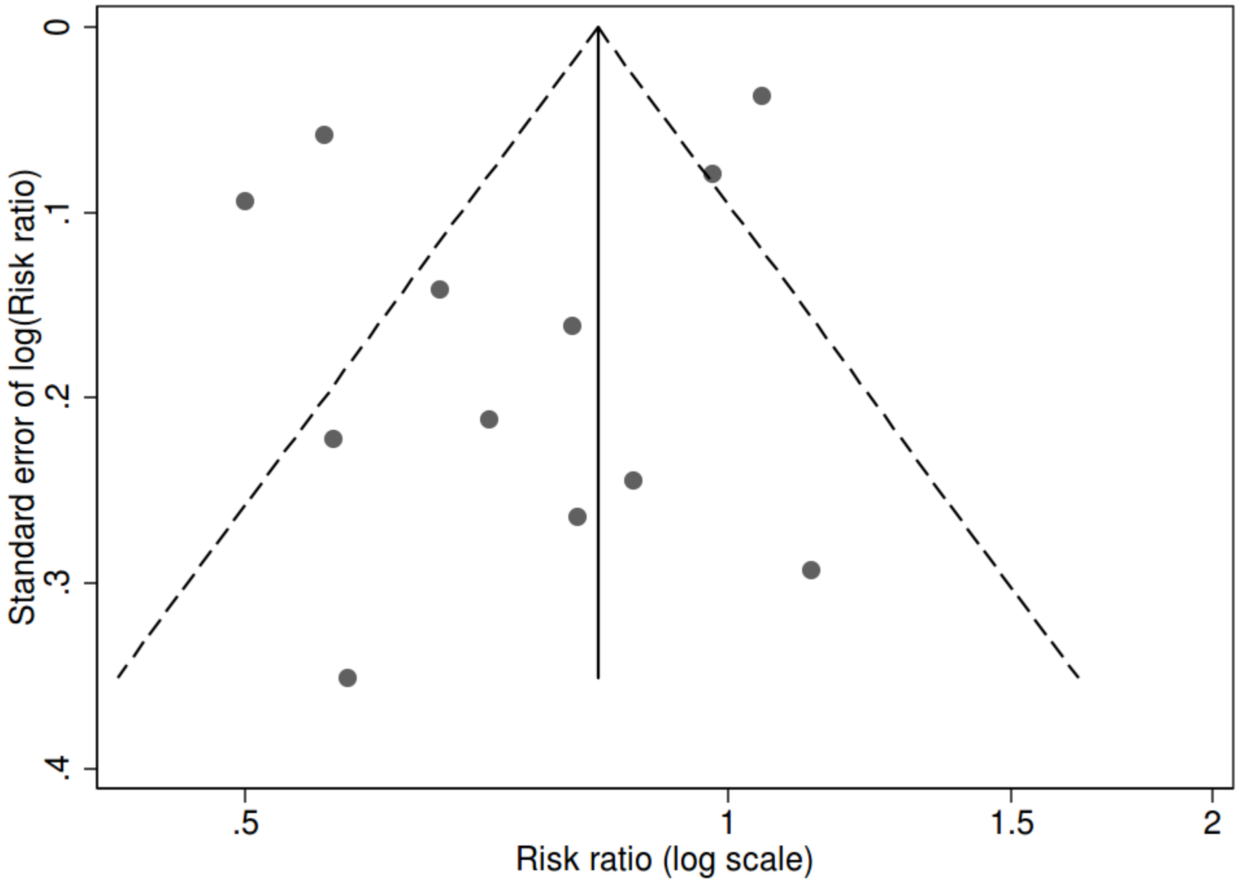

Note: Dotted lines represent pseudo 95% confidence intervals.

Figure S9. Funnel plot of random effects analysis of clinical malaria.

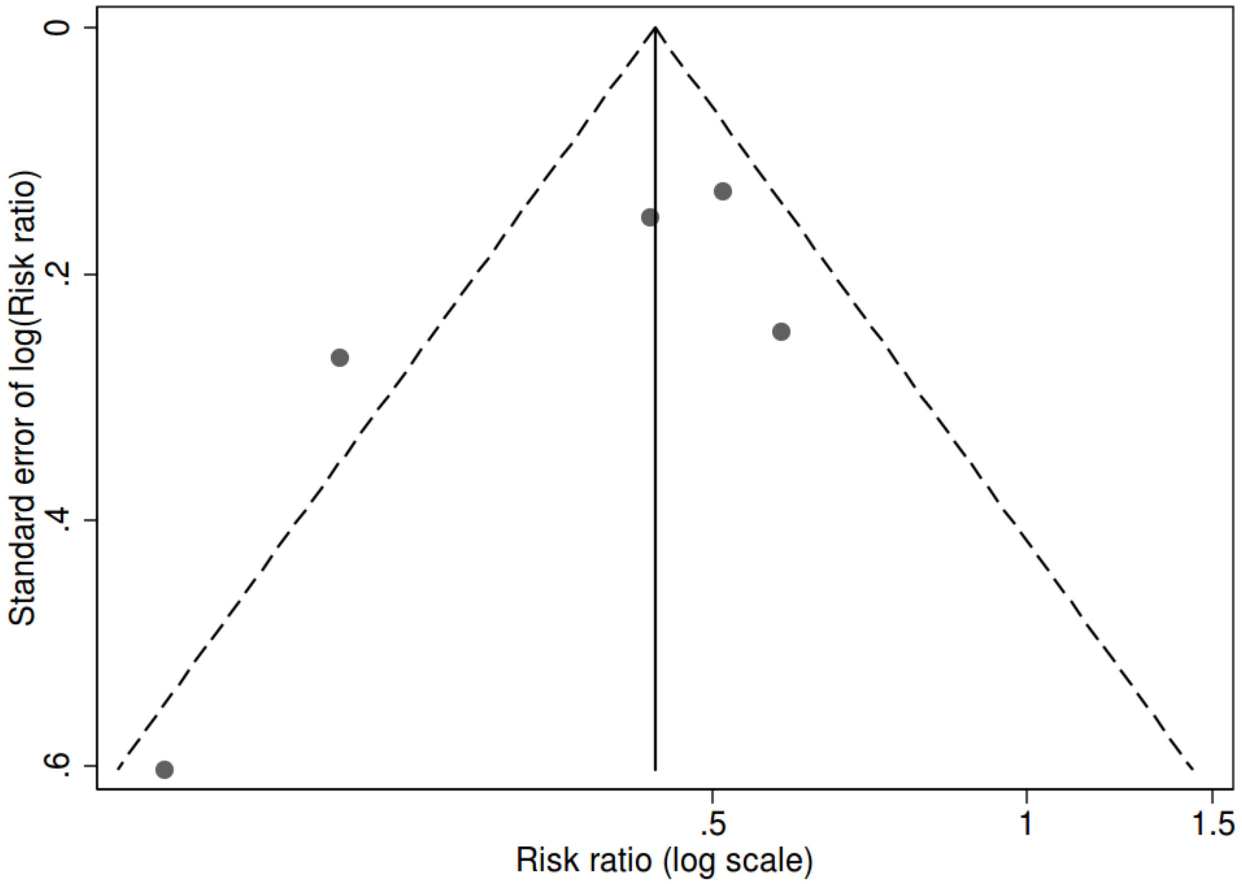

Note: Dotted lines represent pseudo 95% confidence intervals.

Text S1. Search strategy details

Search strategies are provided for searches in PubMed, Embase, Cochrane Central Register of Controlled Trials, and clinicaltrials.gov. Search strategies incorporated both keywords and subject headings. Searches were limited to studies with children as subjects and to articles published since 1990. Searches were tailored to each database due to differences in controlled vocabulary for each database and constraints of the databases.

1. PubMed (pubmed.com)

(asymptomatic[Title/Abstract]) OR subpatent[Title/Abstract] OR carrier[Title/Abstract] OR symptomless[Title/Abstract] OR carrier state[MeSH Terms] OR asymptomatic infections[MeSH Terms])

AND

(malaria[Title/Abstract]) OR parasit*[Title/Abstract] OR plasmodi*[Title/Abstract] OR falciparum[Title/Abstract] OR malaria[MeSH Terms)

AND

(seasonal malaria chemoprophylaxis[Title/Abstract] OR intermittent preventive treatment[Title/Abstract] OR ipt[Title/Abstract] OR intermittent preventive therapy[Title/Abstract]

OR iptc[Title/Abstract] OR iptsc[Title/Abstract] OR intermittent preventive malaria treatment[Title/Abstract] OR chemoprevention[Title/Abstract] OR chemosuppression[Title/Abstract] OR chemoprevention[MeSH Terms] OR antimalarials[Title/Abstract] OR proguanil[Title/Abstract] OR atovaquone[Title/Abstract] OR malarone[Title/Abstract] OR doxycycline[Title/Abstract] OR mefloquine[Title/Abstract] OR lariam[Title/Abstract] OR chloroquine[Title/Abstract] OR primaquine[Title/Abstract] OR aralen[Title/Abstract] OR quinine[Title/Abstract] OR qualaquin[Title/Abstract] OR hydroxychloroquine[Title/Abstract] OR plaquenil[Title/Abstract] OR artemisinin-based combination therapy[Title/Abstract] OR ACT[Title/Abstract] OR artesunate[Title/Abstract] OR Amodiaquine[Title/Abstract] OR Pyrimethamine[Title/Abstract] OR sulfadoxine[Title/Abstract] OR artemisinin[Title/Abstract] OR maloprim[Title/Abstract] OR dapsone[Title/Abstract] OR chlorproguanil[Title/Abstract] OR Lapdap[Title/Abstract] OR Paludrine[Title/Abstract] OR Mepha[Title/Abstract] OR piperaquine[Title/Abstract] OR Fansidar[Title/Abstract] OR antimalarials[MeSH Terms]) OR proguanil[MeSH Terms] OR atovaquone[MeSH Terms] OR "atovaquone, proguanil drug combination"[Supplementary Concept] OR doxycycline[MeSH Terms] OR mefloquine[MeSH Terms] OR chloroquine[MeSH Terms] OR primaquine[MeSH Terms] OR quinine[MeSH Terms] OR hydroxychloroquine[MeSH Terms] OR Artemisinins[MeSH Terms] OR artesunate[Supplementary Concept] OR Amodiaquine[MeSH Terms] OR Pyrimethamine[MeSH Terms] OR sulfadoxine[MeSH Terms] OR Maloprim[Supplementary Concept] OR dapsone[MeSH Terms] OR chlorproguanil[Supplementary Concept] OR chloroguanil, dapsone drug combination[Supplementary Concept] OR clopidogrel[Supplementary Concept] OR piperaquine[Supplementary Concept] OR fanasil, pyrimethamine drug combination[Supplementary Concept]

AND

(child*[Title/Abstract] OR teenager[Title/Abstract] OR adolescen*[Title/Abstract] OR school-age*[Title/Abstract] OR "child"[MeSH Terms] OR "adolescent"[MeSH Terms])

AND

("1990"[Date - Publication] : "3000"[Date - Publication])

2. Embase (embase.com)

(asymptomatic:ab,ti OR subpatent:ab,ti OR carrier:ab,ti OR symptomless:ab,ti)

AND

(malaria:ab,ti OR parasit*:ab,ti OR plasmodi*:ab,ti OR falciparum:ab,ti)

AND

('seasonal malaria chemoprophylaxis':ab,ti OR 'intermittent preventive treatment':ab,ti OR 'ipt':ab,ti OR 'iptC':ab,ti OR 'iptsc':ab,ti OR 'intermittent preventive therapy':ab,ti OR 'intermittent preventive malaria treatment':ab,ti OR chemoprevention:ab,ti OR chemosuppression:ab,ti OR 'chemoprophylaxis'/exp OR antimalarials:ab,ti OR proguanil:ab,ti OR atovaquone:ab,ti OR malarone:ab,ti OR doxycycline:ab,ti OR mefloquine:ab,ti OR lariam:ab,ti OR chloroquine:ab,ti OR primaquine:ab,ti OR aralen:ab,ti OR quinine:ab,ti OR qualaquin:ab,ti OR hydroxychloroquine:ab,ti OR plaquenil:ab,ti OR 'artemisinin-based combination therapy':ab,ti OR act:ab,ti OR artesunate:ab,ti OR amodiaquine:ab,ti OR pyrimethamine:ab,ti OR sulfadoxine:ab,ti OR artemisinin:ab,ti OR maloprim:ab,ti OR dapsone:ab,ti OR chlorproguanil:ab,ti OR lapdap:ab,ti OR paludrine:ab,ti OR mepha:ab,ti OR piperaquine:ab,ti OR fansidar:ab,ti OR 'chemoprophylaxis'/exp OR 'antimalarial agent'/exp OR 'doxycycline'/exp OR 'sulfadoxine'/exp OR 'dapsone plus pyrimethamine'/exp OR 'dapsone'/exp OR 'chlorproguanil plus dapsone'/exp)

AND

(child*:ab,ti OR teenager:ab,ti OR adolescen*:ab,ti OR 'school age':ab,ti OR 'school aged':ab,ti OR 'child'/exp OR 'adolescent'/exp)

AND

[1990-2015]/py

3. Cochrane Central Register of Controlled Trials (Wiley)

(asymptomatic or subpatent or carrier or symptomless)

AND

(malaria or parasit* or plasmodi* or falciparum)

AND

(chemoprophylaxis or chemoprevention or chemosuppression or intermittent preventive or antimalarial*)

AND

(child* or teenage* or adolescen* or school-age*) **LIMIT**: PUBLICATION YEAR FROM 1990

4. Clinicaltrials.gov

asymptomatic malaria OR subpatent malaria OR carrier malaria OR symptomless malaria

Text S2. Creation of variable for proportion of follow-up time protected

The proportion of follow-up time protected by treatment was estimated as the number of days protected by treatment divided by the total number of days between the first dose of treatment and the outcome measurement. Number of days protected by treatment was estimated by multiplying the study drug specific post-treatment chemoprophylaxis period by the number of treatments. Post-treatment chemoprophylaxis period estimates for each drug or drug combination were: dihydroartemisinin-piperaquine 29.4 days[1]; artemether-lumefantrine 13.8 days[1]; artesunate-amodiaquine 14.4 days (ASAQ study group – Okell personal communication); sulfadoxine-pyrimethamine 35 days (mid-point of 4-6 weeks estimated in multiple studies[2–5]); combinations of drugs with shorter half-lives than sulfadoxine-pyrimethamine (artesunate, amodiaquine, piperaquine) when paired with sulfadoxine-pyrimethamine the estimate for sulfadoxine-pyrimethamine was used; primaquine, doxycycline, mefloquine, and chloroquine were not specifically estimated because in the one study that used these drugs the dosing interval (daily or weekly) was designed to be shorter than the drug half-live in order to provide continuous chemoprophylaxis. When specific treatment and outcome dates were not available, the days between the first dose and the outcome measurement were estimated from the mid-point of the month involved. For the outcome clinical malaria in Matangila *et al*, the outcome was measured only in the four months following the first treatment course. However, the proportion of follow-up time protected is the same as for the other outcomes.

References

1. Okell LC, Cairns M, Griffin JT, et al. Contrasting benefits of different artemisinin combination therapies as first-line malaria treatments using model-based cost-effectiveness analysis. *Nat Commun*. 2014;5. doi:10.1038/ncomms6606.
2. Cairns M, Gosling R, Gesase S, Mosha J, Greenwood B, Chandramohan D. Mode of action and choice of antimalarial drugs for intermittent preventive treatment in infants. *Trans R Soc Trop Med Hyg*. 2009;103(12):1199-1201. doi:10.1016/j.trstmh.2009.06.007.
3. Cairns M, Carneiro I, Milligan P, et al. Duration of Protection against Malaria and Anaemia Provided by Intermittent Preventive Treatment in Infants in Navrongo, Ghana. White NJ, ed. *PLoS One*. 2008;3(5):e2227. doi:10.1371/journal.pone.0002227.
4. May J, Adjei S, Busch W, et al. Therapeutic and prophylactic effect of intermittent preventive anti-malarial treatment in infants (IPTi) from Ghana and Gabon. *Malar J*. 2008;7(1):198. doi:10.1186/1475-2875-7-198.
5. Watkins WM, Mberu EK, Winstanley PA, Plowe C V. The efficacy of antifolate antimalarial combinations in Africa: a predictive model based on pharmacodynamic and pharmacokinetic analyses. *Parasitol Today*. 1997;13(12):459-464. http://www.ncbi.nlm.nih.gov/pubmed/15275132. Accessed February 16, 2019.

Text S3. Additional details on coverage in cluster randomized studies

- Coverage is included in Table 1 for cluster randomized studies to show the variation between studies and further interpret the effects of different interventions. Reporting of coverage varies between the studies. The values reported in the table show the closest values available for per-protocol coverage. Coverage estimates are lower in studies in which multiple rounds of treatment were employed. Further details for each study are provided below.

- Clarke et al (2008): The intervention provided treatment on three occasions. 41% of the intervention group received complete treatment.

- Halliday et al (2014): The intervention screened participants in the intervention arm five times. 66.8% of those in the intervention arm were screened for infection in all rounds. On average 88.4% of those in the intervention arm were screened in each round.

- Clarke et al (2017): The intervention provided treatment once. 94.6% of those in the intervention arm received treatment.

- Staedke et al (2019, school-based surveys: personal communication): The intervention provided treatment six times. 7.1% of participants in the intervention arm in the school surveys received treatment in all six rounds. 67% received at least one full treatment dose.
